# Microbiomes of urban trees: unveiling contributions to atmospheric pollution mitigation

**DOI:** 10.3389/fmicb.2024.1470376

**Published:** 2024-11-11

**Authors:** Isabella Gandolfi, Claudia Canedoli, Asia Rosatelli, Stefano Covino, David Cappelletti, Bartolomeo Sebastiani, Valeria Tatangelo, Davide Corengia, Francesca Pittino, Emilio Padoa-Schioppa, Ximena Báez-Matus, Lisette Hernández, Michael Seeger, Zaki Saati-Santamaría, Paula García-Fraile, Rubén López-Mondéjar, Roberto Ambrosini, Maddalena Papacchini, Andrea Franzetti

**Affiliations:** ^1^Department of Earth and Environmental Sciences (DISAT), University of Milano-Bicocca, Milan, Italy; ^2^Department of Chemistry, Biology and Biotechnology, University of Perugia, Perugia, Italy; ^3^Biotreeversity, Carugo, Italy; ^4^Departamento de Química and Centro de Biotecnología Daniel Alkalay Lowitt, Universidad Técnica Federico Santa María, Valparaíso, Chile; ^5^Millenium Nucleus Bioproducts, Genomics and Environmental Genomics (BioGEM), Valparaíso, Chile; ^6^Departamento de Microbiología y Genética, Universidad de Salamanca, Salamanca, Spain; ^7^Instituto de Investigación en Agrobiotecnología (CIALE), Universidad de Salamanca, Salamanca, Spain; ^8^Institute of Microbiology of the Czech Academy of Sciences, Prague, Czechia; ^9^Unidad Asociada Grupo de Interacción Planta-Microorganismo, Universidad de Salamanca-IRNASA-CSIC, Salamanca, Spain; ^10^Department of Soil and Water Conservation and Waste Management, CEBAS-CSIC, Campus Universitario de Espinardo, Murcia, Spain; ^11^Department of Environmental Science and Policy, University of Milan, Milan, Italy; ^12^Department of Technological Innovations and Safety of Plants, Products and Anthropic Settlements, Italian National Institute for Insurance against Accidents at Work (INAIL), Rome, Italy

**Keywords:** urban trees, phyllosphere, tree-related microhabitats, tree cavity organic soil, air pollution mitigation, ecosystem services, hydrocarbon biodegradation

## Abstract

Urban trees are crucial in delivering essential ecosystem services, including air pollution mitigation. This service is influenced by plant associated microbiomes, which can degrade hydrocarbons, support tree health, and influence ecological processes. Yet, our understanding of tree microbiomes remains limited, thus affecting our ability to assess and quantify the ecosystem services provided by trees as complex systems. The main hypothesis of this work was that tree microbiomes concur to hydrocarbon biodegradation, and was tested through three case studies, which collectively investigated two tree micro-habitats (phyllosphere and tree cavity organic soil—TCOS) under various conditions representing diverse ecological scenarios, by applying different culture-based and molecular techniques and at different scales. The integration of all results provided a more comprehensive understanding of the role of microbiomes in urban trees. Firstly, bacterial strains isolated from the phyllosphere of *Quercus ilex* were characterized, indicating the presence of Plant-Growth Promoting bacteria and strains able to catabolize PAHs, particularly naphthalene and phenanthrene. Secondly, naphthalene biodegradation on artificially spiked *Hedera helix* leaves was quantified in greenhouse experiments on inoculated and untreated plants. The persistence of the inoculated strain and community structure of epiphytic bacteria were assessed by Illumina sequencing of V5–V6 hypervariable regions of 16S rRNA gene. Results showed that naphthalene degradation was initially faster on inoculated plants but later the degradation rates became similar, probably because bacterial populations with hydrocarbon-degrading abilities gradually developed also on non-inoculated plants. Finally, we explored bacterial and fungal biodiversity hosted by TCOS samples, collected from six large trees located in an urban park and belonging to different species. Microbial communities were characterized by Illumina sequencing of V5–V6 hypervariable regions of bacterial gene 16S rRNA and of fungal ITS1. Results indicated TCOS as a distinct substrate, whose microbiome is determined both by the host tree and by canopy environmental conditions and has a pronounced aerobic hydrocarbon degradation potential. Overall, a better assessment of biodiversity associated with trees and the subsequent provision of ecosystem services constitute a first step toward developing future new microbe-driven sustainable solutions, especially in terms of support for urban green planning and management policy.

## 1 Introduction

Urban green infrastructures play a fundamental role in creating more sustainable and resilient cities and trees are increasingly seen as a key solution to urban challenges. They are being incorporated into climate-sensitive urban design to improve livability and promote environmental justice (Landry and Chakraborty, 2009). Because of their substantial size and ecological significance, trees play a crucial role in delivering essential urban ecosystem services, including, e.g., carbon sequestration, temperature regulation through shading and transpiration, stormwater management, and the provision of habitats for urban wildlife. Another significant ecosystem service provided by trees in urban areas is air quality regulation (Salmond et al., 2016). Indeed, trees improve air quality through multiple mechanisms: they absorb gaseous pollutants such as ozone, nitrogen dioxide, and sulfur dioxide through the leaf stomata and capture airborne particulate matter on leaves and bark. In addition, the physical structure of trees helps mitigate pollution by reducing wind speed and intercepting dust and particulate matter (Nowak and Heisler, 2010; Grote et al., 2016). However, these services are not solely provided by the physical structure of the trees but probably also by their associated biodiversity. In fact, there is accumulating evidence that microbial communities associated with trees, including bacteria and fungi, can significantly enhance air quality regulation (Perreault and Laforest-Lapointe, 2022; Wei et al., 2017). These microorganisms can be involved in the degradation of organic pollutants, such as hydrocarbons, which are prevalent in urban environments (Dharmasiri et al., 2023; Franzetti et al., 2020; Terzaghi et al., 2021). This bioremediation process could help mitigate air pollution and improve urban air quality, making trees and their microbiomes vital components of urban ecosystems.

The phyllosphere, comprising the aerial parts of plants and dominated by leaves, represents the widest compartment of trees, hosting a variety of organisms, especially bacteria. The global bacterial population present on leaves was estimated to comprise up to 10^26^ cells, corresponding to an average of 10^6^–10^7^ bacteria per cm^2^ of leaf surface (Vorholt, 2012). Many studies have shown the importance of the phyllosphere in air quality regulation, emphasizing its role in the degradation of hydrocarbons (Sangthong et al., 2016; Scheublin et al., 2014; Weyens et al., 2015). However, the relative importance of phyllosphere-located degradation processes compared to other mechanisms of pollution mitigation still needs to be assessed (Perreault and Laforest-Lapointe, 2022).

In addition to the phyllosphere, other tree structures, known as Tree-related Microhabitats (TreMs; Larrieu et al., 2018), provide critical habitats for various species and may therefore contribute to this ecosystem service. TreMs include structures such as cavities, bark crevices, deadwood, and dendrothelms, offering specific substrates and micro-climatic conditions essential for the survival of diverse taxa and are considered hotspots of biodiversity (Martin et al., 2022). These niches are less studied but probably equally important in supporting microbial diversity and ecological functions.

Microorganisms living on trees are collectively referred to as tree microbiomes. They have a long history of co-evolution with trees, forming a holobiont system (Theis et al., 2016). While the rhizosphere has been extensively studied (Cregger et al., 2021; Mercado-Blanco et al., 2018), with a particular focus on mycorrhizae (Chaudhury et al., 2024; Dyshko et al., 2024; Mortier et al., 2020), the phyllosphere and TreMs are promising areas for further investigation. Microbiomes hosted by these habitats potentially play pivotal roles in supporting the health of urban trees and influencing ecological processes, and they may be distinct from those on nearby surfaces (Saeki et al., 2024). However, our understanding of the tree microbiomes remains limited, with much yet to be uncovered regarding their composition, dynamics, and ecological significance. This limitation also affects our ability to assess and quantify the ecosystem services provided by trees as complex systems.

**Figure 1:**
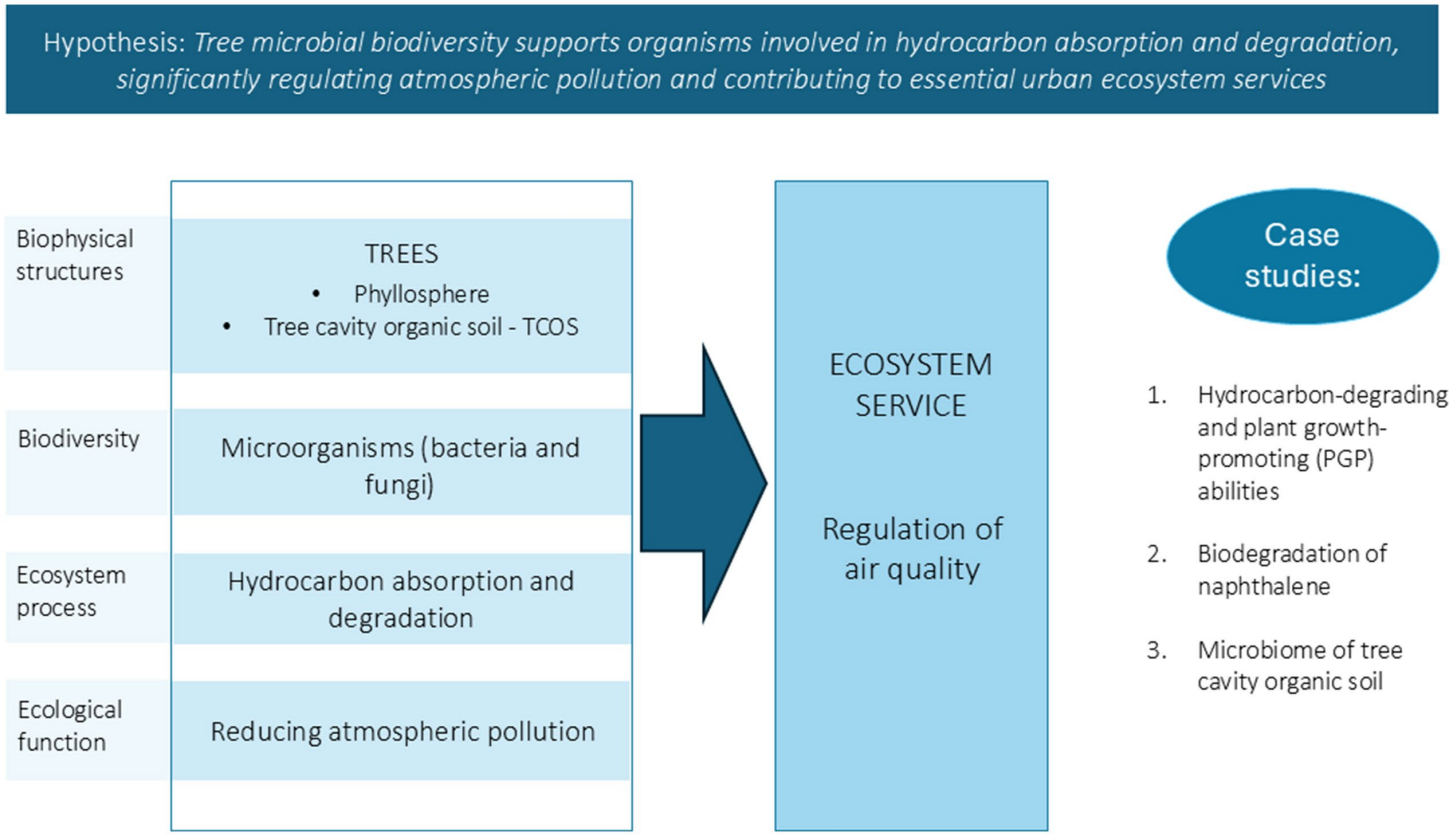
Conceptual model at the basis of this work. Biophysical structures support biodiversity, which interacts with the abiotic environment and regulates ecosystem processes. The figure illustrates how bacteria and fungi hosted by tree leaves and tree cavity soil may absorb and degrade hydrocarbons, reducing atmospheric pollution, and thus providing a crucial ecosystem service that is air quality regulation; the paper tests the hypothesis of tree microbial biodiversity supporting organisms involved in regulation of air pollution through three case studies.

Our research focused on advancing the understanding of microbial tree biodiversity and its role in urban ecosystem services, particularly air quality regulation (Figure 1). In fact, the main hypothesis of this work was that tree microbiomes can collectively concur to ecosystem processes such as absorption of hydrocarbons that are accumulated on the leaves through wet and dry deposition from the atmosphere, and their subsequent degradation, thus playing a critical role in regulating air pollution and contributing to essential urban ecosystem services. To test this, we focused on two tree-hosted habitats: the phyllosphere, due to its huge global area, and tree cavity organic soil (TCOS), to assess how these distinct micro-habitats may contribute to urban air pollution mitigation, especially through hydrocarbon degradation. Although constituting a much smaller volume with respect to the phyllosphere, TCOS represents a promising and interesting microhabitat, since it is ideally perennial. In fact, while the phyllosphere is a typically ephemeral environment, inhabited by microbial communities that, at least on deciduous species, every year undergo new assembly processes while occupying newly formed niches on expanding leaves (Vorholt, 2012), soil in tree cavities can accumulate for much longer times (Remm and Lõhmus, 2011). The persistence of TCOS within tree cavities over extended periods provides a unique ecological niche that may accumulate airborne and soilborne pollutants including hydrocarbons and other xenobiotics. As a result, these micro-environments may become hotspots for the selection and evolution of microbial communities with enhanced abilities to degrade these pollutants, including the persistent ones. Therefore, understanding the composition and functional capabilities of these microbiomes is crucial, as they may act as natural biofilters.

The two selected tree micro-habitats were studied under various conditions representing diverse ecological scenarios, investigating them by different culture-based and culture-independent molecular techniques and at different scales. Particularly, the work was structured in three case studies. Firstly, we screened phyllosphere isolates to study their hydrocarbon-degrading and plant growth-promoting (PGP) abilities, enhancing our understanding of their potential ecological benefits. In fact, detailed information on PGP properties and catabolic abilities of phyllospheric bacteria, although limited to the culturable fraction, can further help evaluate the microbial contribution to ecosystem services involved in air pollution mitigation. We chose to focus on PAH degradation abilities, since they are widespread harmful constituents of urban particulate matter that can be deposited and accumulated on leaves (Franzetti et al., 2020). Secondly, we investigated the biodegradation of naphthalene on ivy (*Hedera helix*) leaves in greenhouse experiments, providing insights into the ability of bacteria-plant systems to cope with hydrocarbon pollution. Lastly, we explored the biodiversity of TCOS, an almost unstudied microhabitat, to uncover the unique microbiomes it hosts and its potential degradation abilities toward hydrocarbons. These case studies collectively contributed to a deeper understanding of tree microbial diversity and functionality in urban environments. Furthermore, we highlight the urgent need to preserve urban trees and their surrounding environments as some means of safeguarding microbial biodiversity and the ecosystem services it sustains.

## 2 Materials and methods

### 2.1 Isolation and characterization of phyllospheric bacterial strains

#### 2.1.1 Sampling

Leaves of holm oak (*Quercus ilex*) were sampled in spring 2016 in a small urban park close to an ARPA (Regional Agency for Environmental Protection) air quality cabin in the city of Terni, Italy (coordinates: 42.560790 N, 12.652011 E), from four different trees (Supplementary Figure S1). This site was chosen as this city and the nearby area are exposed to a severe anthropic pressure given by vehicular traffic and the presence of an industrial hotspot responsible for the emission of both organic and inorganic compounds. Collected leaves were immediately processed.

#### 2.1.2 Isolation and identification of strains

Phyllospheric epiphytic and endophytic bacterial strains were isolated on Plate Count Agar, R2A, King’s B Agar (Oxoid, Basingstoke, UK), and Modified Burk’s Medium-Agar (VanInsberghe et al., 2013), as follows. Leaves were first immersed into MgSO_4_ 10 mM, vortexed for 3 × 1 min and sonicated for 3 × 1 min to detach epiphytic bacteria; the obtained solution was serially diluted and plated. The same leaves were then rinsed three times with water, submerged in NaClO 1% for 3 min, and rinsed again three times with water to sterilize their surface. Leaf pieces were then placed in a mortar and thoroughly grinded to release endophytic bacteria. The obtained mash was serially diluted with MgSO_4_ 10 mM and plated. Plates were incubated at 10°C or 25°C for up to 14 days; the two incubation temperatures were chosen to roughly represent average winter and summer temperatures, respectively, recorded in Terni in 2016 (https://www.regione.umbria.it/ambiente/servizio-idrografico). Identification of the isolates was carried out through amplification and sequencing of 16S rRNA gene with primers 8F (5′-AGA GTT TGA TCC TGG CTC AG-3′) and 1507R (5′-GGA TCC TAC CTT GTT ACG ACT TCA CCC CAG-3′) (Federici et al., 2011). The obtained sequences were taxonomically classified through the MEGABlast tool (NCBI).

#### 2.1.3 Screening for PAH-degrading bacteria

PAH degradation ability of the isolates was tested in minimal medium Bushnell-Haas (BH) Agar supplemented with thiamine 0.01 mg L^−1^ and oligo-elements (Supplementary Table S1). The first screening was run in Petri dishes, using naphthalene and phenanthrene crystals placed on the lid as the only carbon and energy source. Isolates showing very strong growth in the first screening were also tested in BH liquid medium to assess PAH utilization. This second screening was carried out in 100 mL Erlenmeyer flasks. PAH stock solutions were prepared in acetone at the following concentrations: 20 g L^−1^ for naphthalene, 4 g L^−1^ for 3 ring-PAHs (phenanthrene and anthracene), 4 g L^−1^ for 4 and 5 ring-PAH mixture (fluoranthene, pyrene, benzo[a]anthracene, chrysene, benzo[a]pyrene). Microbial inocula were grown in liquid Nutrient Broth (NB) diluted 1:10 in physiological solution. To remove any growth medium residue the bacterial biomass was repeatedly washed with an isotonic solution (Ringer’s Solution ¼ strength) and re-suspended in 7 mL BH liquid medium at a density of 1 × 10^6^ CFU mL^−1^. Tests in BH liquid medium were set up by adding an aliquot of 50 μL of PAH stock solutions in each flask to reach final concentrations of 50 μg mL^−1^ for naphthalene (3 replicates per isolate), 10 μg mL^−1^ for 3 ring-PAHs (6 replicates per isolate), 10 μg mL^−1^ for each compound in the 4–5 ring-PAH mixture (6 replicates per isolate). The flasks were left open in a laminar flow cabinet for 3–5 min to let the solvent evaporate, then 19 mL of liquid BH medium and 1 mL of bacterial inoculum were added in each flask. Heat-killed control flasks were also set up to evaluate PAH losses by abiotic decomposition and losses due to the extraction process, while biotic controls were prepared with BH medium and bacterial inoculum only, to confirm lack of microbial growth due to the absence of the carbon source. All flasks were incubated in an orbital shaker (200 rpm) at 25°C for different time periods: 24 h for the flasks containing naphthalene, and 3 days or 7 days for flasks containing 3 ring-PAHs or 4–5 ring-PAH mixture.

At the end of each incubation assay, culture broths were extracted with 3 × 10 mL of dichloromethane (DMC). NaCl (0.9% w/v) and 20 μL of HCl 3 M were added with the first aliquot of DMC to reduce formation of emulsions and to acidify the medium. To remove any aqueous residue, the extracts were passed through mini-columns containing anhydrous sodium sulfate (NaSO_4_). Quantitative analysis of the extracts was carried out using a GC mod. 7890A (Agilent Technologies, United States) equipped with a VF-5MS column (15 m × 0.15 mm × 0.15 μm + 3 m EZ-Guard; Agilent J&W) and coupled to a mass analyzer mod 5975C -VL MSD with Triple-Axis Detector (Agilent Technologies, United States), operating in single ion monitoring (SIM). The same instrumental conditions were used to quantify naphthalene in the biodegradation experiments on ivy leaves (see section 2.2.3 below).

Results were expressed as percentage of PAH removal (naphthalene, phenanthrene, anthracene) or of PAH residual concentration (4–5 ring-PAHs), with respect to the heat-killed controls. Differences between strains in PAH degradation and among PAH degradation rates for each strain were assessed through non-parametric (Kruskal-Wallis) tests followed by Dunn post-hoc tests.

#### 2.1.4 Assessment of plant growth promotion traits

Nine strains, selected as representative of genera that are most commonly described as members of phyllosphere microbiomes by more comprehensive molecular 16S rRNA-based surveys (Bulgarelli et al., 2013; Delmotte et al., 2009; Rastogi et al., 2013; Vorholt, 2012), were screened for their Plant-Growth Promotion (PGP) properties (Table 1).

**Table 1:** List of the phyllosphere isolated strains considered in the present study and their identification based on the best-hit against 16S rRNA sequences of type strains (NCBI database).

Strain	Origin (epi-endophyte)	Culture medium of isolation	Closest type strain (% identity)
Is.2^*^	Epiphyte	PCA	*Methylobacterium adhaesivum* (97%)
**Is.4** ^*^	**Epiphyte**	**PCA**	***Methylobacterium goesingense* (99%)**
Is.30^†^	Epiphyte	PCA	*Curtobacterium flaccumfaciens* (98%)
Is.35^*^	Epiphyte	PCA	*Curtobacterium oceanosedimentum* (97%)
**Is.42** ^*^	**Epiphyte**	**R2A**	***Methylobacterium goesingense* (99%)**
Is.63^*^	Epiphyte	R2A	*Massilia aurea* (100%)
**Is.103** ^*^	**Endophyte**	**R2A**	***Sphingomonas yunnanensis* (99%)**
Is.107^*^	Endophyte	R2A	*Aeromicrobium fastidiosum* (99%)
**Is.112**	**Endophyte**	**R2A**	***Sphingomonas endophytica* (98%)**
**Is.194**	**Epiphyte**	**MBM**	***Methylobacterium marchantiae* (99%)**
**Is.202** ^*^	**Epiphyte**	**MBM**	***Sphingomonas panni* (98%)**
Is.289^*†^	Endophyte	R2A	*Rhizobium soli* (99%)

Strains included in the second phase of screening, conducted to analyze their degradation capabilities toward PAHs, are indicated in bold. Strains for which PGP properties were studied are indicated with an asterisk (*). Strains used as inoculum for ivy leaves are indicated with the symbol †.

Phosphate solubilization was evaluated using Pikovskaya medium (Sundara Rao and Sinha, 1963) containing 0.2% calcium phosphate (CaHPO_4_) as P source. Moreover, NBRIP medium (Nautiyal, 1999) containing 0.2% hydroxyapatite (Ca_5_(OH)(PO_4_))_3_ as P source was employed to confirm the results obtained in the previous test. For both tests, three replicates for each strain were incubated at 28°C and phosphate solubilization was detected by the presence of clear halos surrounding the colonies (Jiménez-Gómez et al., 2018).

Siderophore production was analyzed in M9-CAS-Agar medium (Schwyn and Neilands, 1987) modified with the addition of hexamethylenediaminetetraacetic acid (HMDTA), which stabilizes the Fe-CAS complex and works as a color indicator (Alexander and Zuberer, 1991), providing the medium its blue color and characteristic orange halos when siderophores are produced (Jiménez-Gómez et al., 2018). The medium was also supplemented with a chelating agent (EDTA). Three replicates for each strain were incubated at 28°C for 60 days. To monitor siderophore production, the appearance and development of halos were periodically checked for the whole incubation period.

Potassium solubilization was assessed on plates containing Aleksandrov medium modified by Parmar and Sindhu (2013). Three replicates for each strain were incubated at 28°C for 7 days. Potassium solubilization was inferred by the presence of solubilization halos, whose evolution was checked daily for the whole incubation period (Poveda et al., 2019).

Auxin production was tested in JMM medium (O’Hara et al., 1989) supplemented with 0.167 g L^−1^ of tryptophan used as an inductor for auxin synthesis (García-Fraile et al., 2012). Bacterial cultures were incubated at 28°C for 5 days in complete darkness. After the incubation period, 1 mL of bacterial suspension was centrifuged at 10,000 rpm for 3 min. The supernatant was recovered and 500 μL were supplemented with 1 mL of Salkowsky reagent. Auxin production caused toning of the replicates toward a pink-red color induced by the presence of the Salkowsky reagent. Moreover, auxin production was quantified through the intensity of the pink-red color by spectrophotometry at 550 nm using an ATI Unicam 8625 Spectrometer (Mattson®, United States) (García-Fraile et al., 2012). Three replicates of each strain were analyzed to average the results.

ACC deaminase production was evaluated in a minimal medium containing 0.3 g L^−1^ K_2_HPO_4_, 0.3 g L^−1^ KH_2_PO_4_, 0.15 g L^−1^ MgSO_4_^.^7H_2_O, 0.05 g L^−1^ CaCl_2_^.^2H_2_O, 0.1 g L^−1^ NaCl, supplemented with 0.1 g L^−1^ NH_4_NO_3_ and/or 10 g L^−1^ glucose when required (Supplementary Table S2). The medium was also supplemented with ACC (1-aminocyclopropane-1-carboxylic acid) to achieve a 3.0 mM final concentration (Penrose and Glick, 2003). Seven different conditions were tested, for each isolate, to assess the ability of the examined strains to use ACC as carbon source, as nitrogen source or both (Supplementary Table S2). All cultures were incubated at 20°C for 15 days at 180 rpm.

Cellulase production was evaluated using Mateos and colleagues’ protocol (Mateos et al., 1992) which establishes growth inside a double layer plate containing Nutrient Agar (NA) in the lower section and carboxymethylcellulose-agar (CMC-agar) in the upper layer. A solution of 5 mL of 0.1% Congo Red was poured on the agar surface to observe bleaching halos caused by the bacterial cellulolytic activity (Peral-Aranega et al., 2020). The same dye was used to determine the ability to synthesize cellulose as carbon stock (Robledo et al., 2012). Positive samples developed a red coloring. The intensity of the staining was proportional to the production of cellulose.

### 2.2 Biodegradation of naphthalene on ivy leaves

#### 2.2.1 Preliminary tests on *Hedera helix*

Both preliminary tests and naphthalene degradation assays (section 2.2.2) were performed on common ivy plants purchased from the same garden center. Unless otherwise specified, before and during the experiments, plants were kept in a non-heated room, connected to the outside, in controlled conditions of light (12 h dark/12 h light).

##### 2.2.1.1 Leaf spiking with naphthalene

Leaves of *H. helix* plants were manually spiked with naphthalene dissolved in acetone (1 mg L^−1^). The absence of visible damage due to acetone contact with leaves was verified in advance (data not shown). The homogeneity of naphthalene spiking was verified as follows: before contamination, 4–5 leaves were sampled from two different *H. helix* plants to measure the basal level of naphthalene on the leaves; the plants were then spiked with a single spray of the 1 mg L^−1^ solution per leaf, with the aid of a plastic sprayer under a chemical hood. After letting the leaves dry, two samples (4–5 leaves) per plant were collected, one from the top of the plant and one from the bottom. Naphthalene concentration was quantified as reported in 2.2.3.

##### 2.2.1.2 Leaf sterilization

To obtain an abiotic control for naphthalene degradation in subsequent experiments, a 1% NaClO solution was used to treat both the upper and lower parts of the leaves with the aid of a sponge. To preliminarily test the validity of the treatment, a plate count was carried out on a sample of untreated leaves and a sample of treated leaves. Two samples of 5 leaves each were then taken from an ivy plant, before and after the treatment, respectively; serial dilutions were carried out in MgSO_4_ 10 mM, plated on LD agar medium, and incubated at 30°C for 24–48 h.

##### 2.2.1.3 Leaf inoculation

Three different bacterial strains from laboratory collections of phyllospheric isolates, able to degrade naphthalene as sole carbon and energy source, were selected for plant experiments and inoculated on leaves through the foliar spray technique (Preininger et al., 2018). Two of them belonged to the strain collection obtained as described in section 2.1 (Is. 30—*Curtobacterium flaccumfaciens* and Is. 289—*Rhizobium soli*; Tables 1, 2), while the third one (*Sphingomonas* sp.) was retrieved from a different collection. Isolates were inoculated in 20 mL of liquid LD medium (tryptone 10 g L^−1^, yeast extract 5 g L^−1^, NaCl 5 g L^−1^) and grown overnight at 30°C under shaking. Cell cultures were centrifuged for 10 min at 5,000 g and the pellet was resuspended in 75 mL of minimal M9 medium. Inocula were then applied to ivy plants by using a common garden sprayer and spraying twice for each leaf. Two plants were inoculated with each strain. Furthermore, since wetting agents have been indicated as useful to improve wettability and adhesion of the spray on leaves that are typically hydrophobic (Preininger et al., 2018), we also tested a different formulation for one inoculum, by including a surfactant in the bacterial suspension. Therefore, two more plants were inoculated with *Sphingomonas* sp. resuspended in M9 medium added with 0.1% Tween 20. To evaluate the persistence of the bacteria inoculated on the leaves, 3–4 leaves were sampled from each plant at *t* = 0 and after 1, 2, 4, and 8 weeks. DNA of epiphytic microorganisms was extracted from leaf samples, and community composition was assessed through Illumina sequencing of a fragment of 16S rRNA gene, as described in section 2.2.4.

**Table 2:** Results of the tests conducted on *Q. ilex* phyllospheric isolated strains to assay their plant growth promoting activity.

	Phyto-hormone modulation	Stress ethylene reduction	Phosphate solubilization		Potassium solubilization	Metal uptake	Synthesis/degradation of plant macropolymers	
Strains	Auxin production	ACC-deaminase production	Pikovskaya medium	NBRIP medium	Aleksandrov medium	Siderophore production	Cellulase production	Cellulose biosynthesis
Is.2	**−**	**−**	**w**	+	**−**	+	++	+
Is.4	+	**−**	**w**	**w**	**−**	**NG**	++	+
Is.35	**w**	**−**	++	**NG**	**NG**	**NG**	**−**	+
Is.42	**w**	**−**	**−**	**−**	**−**	**NG**	+	+
Is.63	++	**−**	**−**	**−**	**−**	**NG**	++	+
Is.103	**−**	**−**	**−**	**−**	**NG**	**NG**	**w**	+
Is.107	+++	**−**	+	**−**	**−**	**NG**	**w**	+
Is.202	+	**−**	**−**	**−**	**−**	**NG**	+	+
**Is.289**	+	**−**	++	++	**NG**	**NG**	+	+

Qualitative results are expressed as follows: NG = no growth; − = negative; w = weakly positive; + = positive; ++ = very positive; +++ = outstanding. The isolate indicated in bold was also tested as inoculum on ivy leaves, as described in section 2.2.

##### 2.2.1.4 Assessment of naphthalene abiotic loss

To test for the possible abiotic loss of naphthalene from the leaf surface of plants, two plants were treated with NaClO 1% as described in section 2.2.1.2 to minimize microbial activity. A leaf sample (approximately 2 g leaves) was collected from each plant at 0, 6, 24, 48, and 96 h to measure naphthalene concentration on leaves as described in section 2.2.3. During the experiment, plants were kept in a close plexiglass greenhouse to reduce naphthalene sublimation and bacterial contamination from external sources. The experiment was conducted in early summer (June), at an average temperature of 24.0°C.

#### 2.2.2 Naphthalene biodegradation in greenhouse experiments

Naphthalene degradation test was set up with 6 *H. helix* plants, two of which were inoculated with *Sphingomonas* sp. resuspended in M9 medium only as described in section 2.2.1.3, two were sterilized with 1% NaClO as described in section 2.2.1.2, and two were controls without any treatment. All plants were spiked with 1 mg L^−1^ naphthalene in acetone solution as described in section 2.2.1.1; for the inoculated plants, spiking was carried out before inoculation. The experiment was conducted in late summer, between the end of August and the beginning of September, at an average temperature of 22.5°C. A leaf sample (approximately 2 g leaves) was taken from each plant for chemical analysis immediately after spiking (after foliar spray for inoculated plants), and after 2, 4, 8 and 16 days. Chemical analyses were performed as described in section 2.2.3. Another leaf sample (3–4 leaves if inoculated, 5–6 leaves if not inoculated) was taken from each plant at the same time for the microbiological analyses (only at 16 days for sterilized plants to evaluate a potential bacterial recolonization of the leaves). DNA of epiphytic microorganisms was extracted from these leaf samples, and community composition was assessed through Illumina sequencing of a fragment of 16S rRNA gene, as described in section 2.2.4.

#### 2.2.3 Chemical analyses

Naphthalene was extracted from *H. helix* leaves of various sizes, with areas ranging from 30 to 60 cm^2^, as estimated from leaf images using ImageJ software (NIH, United States). The leaves were washed twice for 1 min in dichloromethane (2 × 30 mL) under mechanical agitation at 400 rpm. Before the extraction, the samples were spiked with a solution of perdeuterated naphthalene as an internal standard for quantification. The extracts were concentrated, dissolved in hexane and purified in a 3% w/w H_2_O deactivated silica gel column (70–230 mesh ASTM, Merck) for the subsequent analysis. Naphthalene was quantified according to the GC–MS conditions described in section 2.1.3. Differences in degradation efficiency were assessed using a linear model on the log-transformed residual concentrations of naphthalene over time and experimental conditions (three-level factor). The intercept was set at 100% at the starting time for all experimental groups.

#### 2.2.4 Microbiological molecular analyses

To extract DNA of epiphytic microorganisms, sampled leaves were put in a 50-mL tube with 10 mL of washing solution (Tris–HCl pH 7.2 20 mM; EDTA pH 8 10 mM; Tween 20 0.1%). After vortexing for 4 min leaves were discarded. Solid particles contained in the washing solution were collected as follows: a 2 mL aliquot of the solution was centrifuged in a 2-mL tube for 2 min at 10,000 g. After the centrifugation, the supernatant was removed with a micropipette, being careful to avoid the pellet, and another 2 mL of the remaining solution were added to the same tube. The same steps of centrifugation and supernatant discarding were repeated until all the solution was processed. The pellet was then resuspended in 978 μL of Sodium Phosphate Buffer of FastDNA^™^ Spin Kit for Soil (MP Biomedicals, Solon, OH, United States), and tube content was transferred into a Lysing Matrix E tube of the same kit (modified from Smets et al., 2022). Total DNA was then extracted following the manufacturer’s instructions of the kit. The V5–V6 hypervariable regions of the bacterial 16S rRNA gene were PCR-amplified using 783F and 1046R primers, as described in Nava et al., 2024, except that 60-μL volume PCR reactions were prepared due to the averagely low DNA amount obtained from epiphytic microorganisms. Amplicons were sequenced by MiSeq Illumina (Illumina, Inc., San Diego, CA, USA) with a 2 × 300 bp paired-end protocol. Amplicon Sequence Variants (ASVs) were inferred using the DADA2 pipeline (Callahan et al., 2016), with trimming of the forward and reverse reads after 180 and 150 bases, respectively, and quality filtering with a maximum number of expected errors equal to 0.5 per read. The two reads were then merged and taxonomically assigned with SILVA v132 databases. All non-bacterial ASVs, i.e., those belonging to Archaea or Eukarya domains, those that were not classified at domain level, and those classified as chloroplasts or mitochondria, were discarded. The remaining ASVs were classified again with the latest version of the RDP classifier (ver. 2.14, August 2023).

#### 2.2.5 Estimation of hydrocarbon degrading potential from genus abundance table

To estimate the relative abundance of bacterial populations classified at the genus level and possessing selected hydrocarbon-degrading genes, the original relative abundance of each genus was multiplied by a coefficient ranging from 0 to 1, representing the estimated fraction of a certain genus which harbors the marker genes relevant for aerobic and anaerobic degradation processes of aliphatic and aromatic hydrocarbons. To obtain these coefficients, the Hidden Markov Models of the main marker genes for hydrocarbon degradation were searched (hmmseach command) against the GTDB genome database (Khot et al., 2022; Parks et al., 2022). This method was previously applied to infer potential degradation abilities especially in underexplored environments, typically marine, but also in petroleum reservoirs and forest soils (see, e.g., Calderon-Fajardo et al., 2024; Góngora et al., 2024; Howe et al., 2024; Nelson et al., 2024).

### 2.3 Microbial diversity in tree cavity organic soil

#### 2.3.1 Sampling

TCOS samples were collected from six mature trees belonging to frequently occurring species in urban areas (common oak, *Quercus robur*; deodar cedar, *Cedrus deodara*; southern magnolia, *Magnolia grandiflora*; small-leaved lime, *Tilia cordata*; London plane tree, *Platanus acerifolia*; Caucasian walnut, *Pterocarya fraxinifolia*) located in a central urban park (Giardini Indro Montanelli, 45.4744912, 9.2003923) in Milan, Italy (Supplementary Figure S2). Sampling was performed in November 2022 (autumn) and April–May 2023 (spring) to assess possible seasonal variations in microbial communities. To explore the entire vertical and horizontal structure of trees, samples were collected by tree climbers, whenever cavities containing TCOS were reachable by the operator. A total of 20 samples could be collected (12 in autumn and 8 in spring; Table 3).

**Table 3:** List of collected tree cavity organic soil (TCOS) samples.

Code	Tree species	Cavity height (m)	Season
Cedar-1a-AU	*Cedrus deodara*	22	Autumn
Cedar-1b-AU	*Cedrus deodara*	22	Autumn
Magnolia-1-AU	*Magnolia grandiflora*	15	Autumn
Walnut-1-AU	*Pterocarya fraxinifolia*	22	Autumn
Walnut-2-AU	*Pterocarya fraxinifolia*	8	Autumn
Walnut-3-AU	*Pterocarya fraxinifolia*	5	Autumn
Walnut-3b-AU	*Pterocarya fraxinifolia*	5	Autumn
Walnut-4-AU	*Pterocarya fraxinifolia*	2.5	Autumn
Plane_tree-1-AU	*Platanus acerifolia*	22	Autumn
Plane_tree-2-AU	*Platanus acerifolia*	15	Autumn
Oak-1-AU	*Quercus robur*	25	Autumn
Lime-1-AU	*Tilia cordata*	7	Autumn
Walnut-1a-SP	*Pterocarya fraxinifolia*	8	Spring
Walnut-1b-SP	*Pterocarya fraxinifolia*	8	Spring
Walnut-1c-SP	*Pterocarya fraxinifolia*	8	Spring
Oak-1-SP	*Quercus robur*	4	Spring
Magnolia-1a-SP	*Magnolia grandiflora*	2	Spring
Magnolia-1b-SP	*Magnolia grandiflora*	2	Spring
Lime-1a-SP	*Tilia cordata*	12	Spring
Lime-1b-SP	*Tilia cordata*	12	Spring

Different numbers for the same tree in the same season indicate different cavities from which TCOS was sampled. Lower-case letters next to the same number indicate replicates of TCOS sampled from the same cavity.

#### 2.3.2 DNA extraction, sequencing and sequence analysis

Total DNA was extracted from 0.5 g of TCOS using the FastDNA^™^ Spin Kit for Soil (MP Biomedicals, Solon, OH, United States), according to manufacturer’s instructions. Bacterial communities were characterized through PCR amplification of the V5–V6 hypervariable regions of 16S rRNA gene, as described in Nava et al. (2024). Fungal communities were characterized through PCR amplification of the ITS1 region using the ITS1F/ITS2R primer pair (Buée et al., 2009), with 6-bp barcodes added at the 5′-end to allow sample pooling and sequence sorting. For each sample, 2 × 50 μL PCR reactions were prepared with GoTaq^®^ G2 Green Master Mix (Promega Corporation, Madison, WI, United States) and 1 μM of each primer. The cycling conditions were as follows: initial denaturation at 94°C for 4 min; 30 cycles of 94°C for 30 s, 50°C for 30 s, and 72°C for 1 min; final extension at 72°C for 5 min. The amplicons were purified using the Wizard^®^ SV Gel and PCR Clean-up System (Promega Corporation, Madison, WI) and their quantity was determined using the Qubit^®^ fluorometer (Life Technologies, Carlsbad, CA). All amplicons were sequenced by MiSeq Illumina (Illumina, Inc., San Diego, CA, United States) with a 2 × 300 bp paired-end protocol. For bacterial communities, Amplicon Sequence Variants (ASVs) were inferred as described in section 2.2.4. For fungal communities, the forward read only (R1) was considered for inferring ASVs (Pauvert et al., 2019), which were taxonomically assigned with the latest version of the UNITE database (RDP classifier ver. 2.14, August 2023). Data distribution was visualized by performing separate Principal Component Analyses (PCA) on bacterial and fungal communities. PCAs were carried out on ASV tables and were based on Hellinger distances with the Vegan package in R. The hydrocarbon degradation potential of bacterial communities was predicted as described in section 2.2.5.

## 3 Results

### 3.1 Isolation and characterization of phyllospheric bacterial strains

#### 3.1.1 Isolation and identification of strains

From the phyllosphere of *Q. ilex*, a total of 374 bacterial strains were isolated, which were tested for their hydrocarbonoclastic abilities in minimal medium Bushnell Hass (BH) Agar supplemented with naphthalene and phenanthrene crystals. Overall, 10.2% of isolates (38 strains) were able to use naphthalene as the sole carbon source, while 9.1% (34 strains) were able to grow on phenanthrene; they mainly belonged to genera *Sphingomonas, Methylobacterium, Bacillus*, and *Rathayibacter* (Supplementary Table S3). Interestingly, most (33) of these strains were able to degrade both naphthalene and phenanthrene, while only 5 strains grew exclusively on naphthalene and one strain exclusively on phenanthrene. Following this pre-screening and identification through 16S rRNA amplification, 15 isolates were chosen to conduct further analysis for Plant Growth Promoting abilities and, among those, 6 strains were studied thoroughly for their degradation capacity toward naphthalene, 3 ring-PAHs (phenanthrene and anthracene) and 4–5 ring-PAHs (fluoranthene, pyrene, benzo[a]pyrene, chrysene, benzo[a]anthracene) (Table 1).

#### 3.1.2 Screening for PAH-degrading bacteria

**Figure 2:**
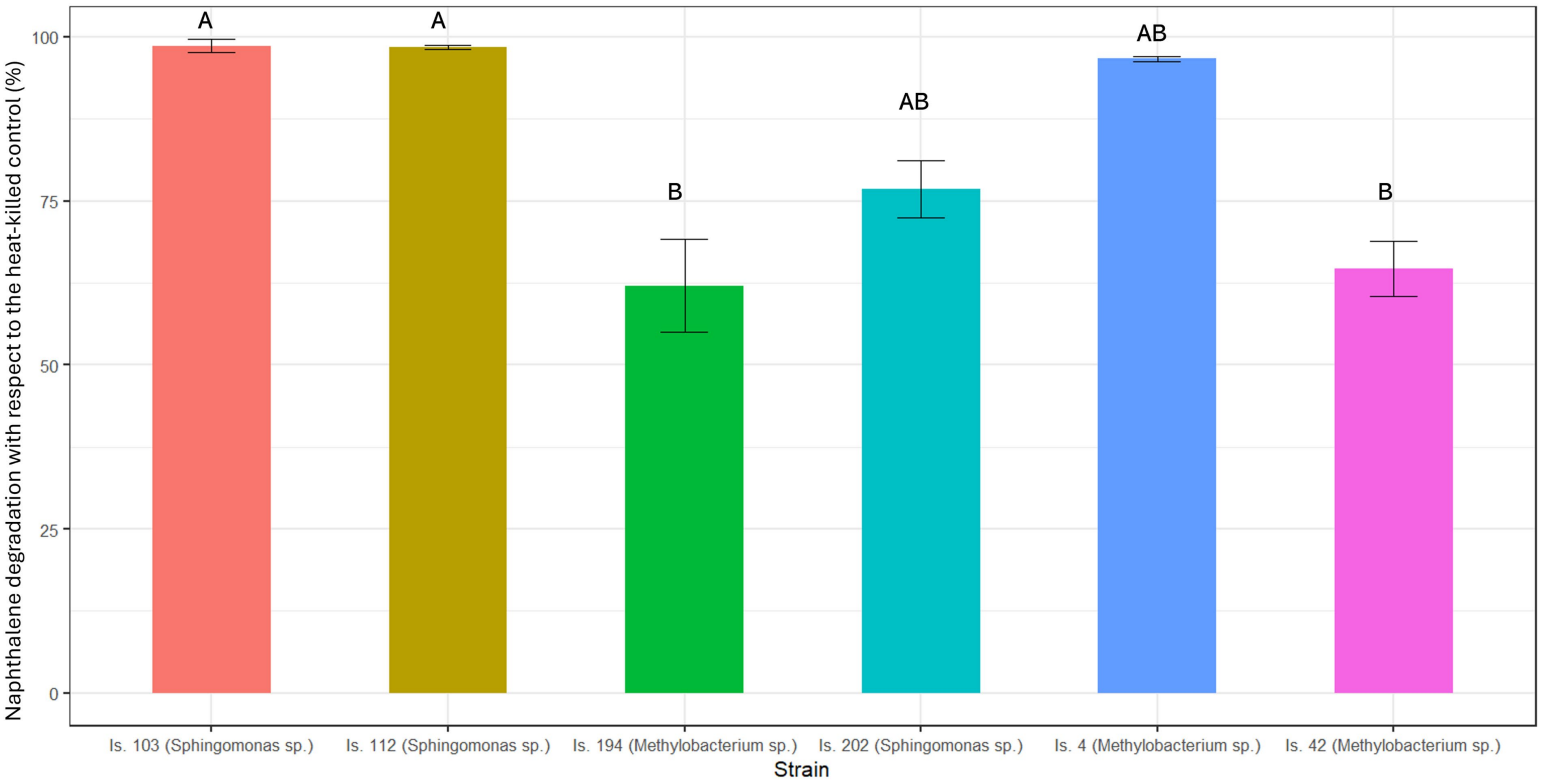
Naphthalene degradation after 24 h incubation at 25°C. Results are presented as mean ± standard deviation of the three replicates for each experimental condition. Identical letters indicate the lack of a significant difference.

Due to its high volatility, naphthalene degradation tests were carried out for 24 h only. During the incubation period, naphthalene abiotic loss accounted for 35 ± 5% SE of the initial concentration. The isolates showed large and statistically significant differences in their naphthalene degradation efficiency (Kruskal-Wallis tests: χ^2^_5_ = 18.36, *p* = 0.003). As shown in Figure 2, two isolates, belonging to the genus *Sphingomonas* (Is.103 and Is. 112), showed a significantly higher efficiency in catabolizing naphthalene (>97% removal with respect to the heat-killed control) than Is. 194 and Is. 42, both belonging to the genus *Methylobacterium* (|*z*| ≥ 2.74, *p* ≤ 0.023). Is. 202 (*Sphingomonas* sp.) and Is. 4 (*Methylobacterium* sp.) achieved an intermediate removal efficiency of naphthalene (77 ± 2% SE and 96 ± 0.3% SE), which did not differ significantly from the other isolates (|*z*| ≤ 1.79, *p* ≥ 0.156). Is. 194 and Is. 42 (*Methylobacterium* sp.) reached 62 ± 4% SE and 65 ± 2% SE naphthalene removal, respectively.

**Figure 3:**
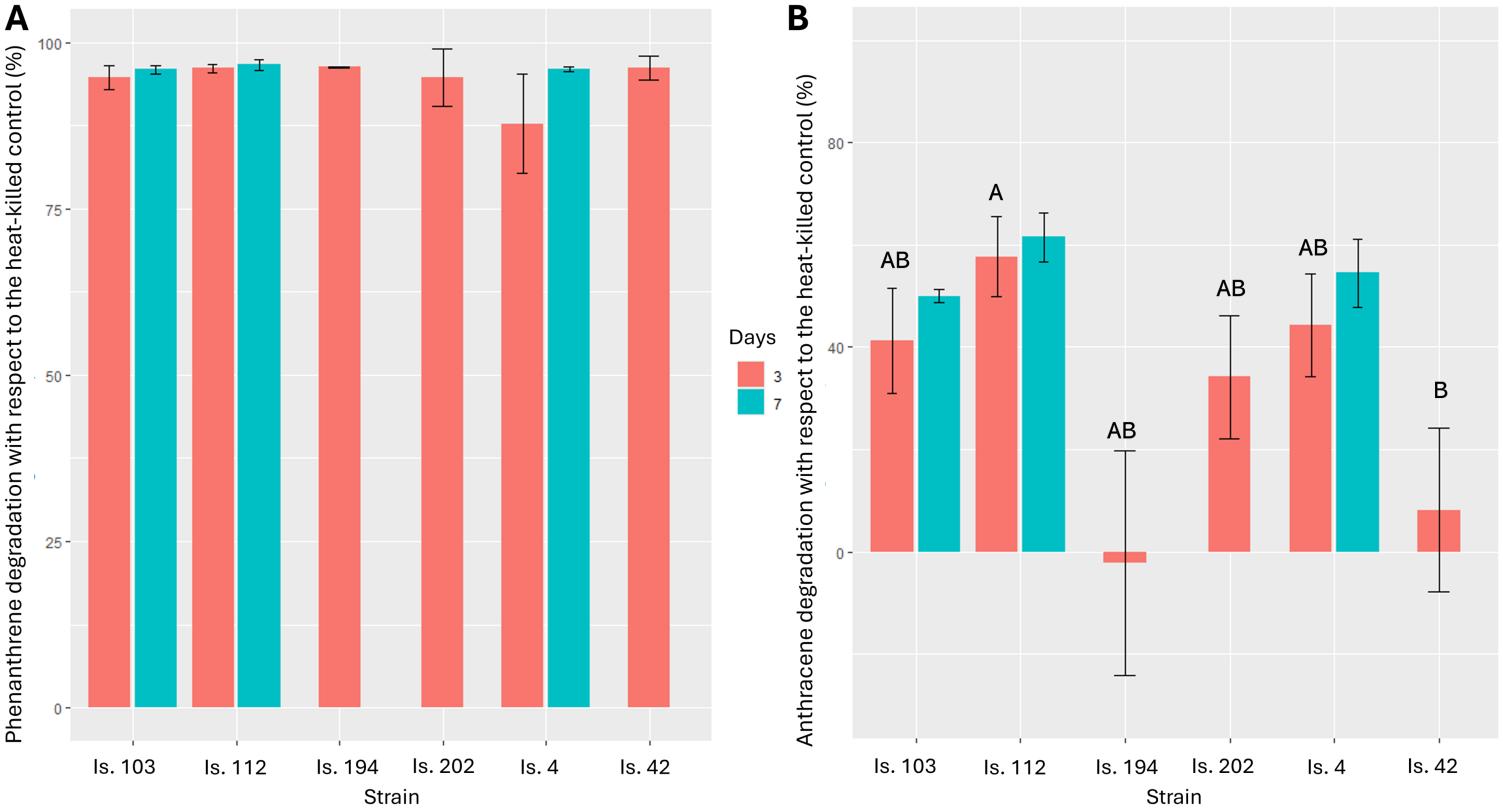
Phenanthrene (A) and anthracene (B) degradation after 3 and 7 days of incubation at 25°C. Results are presented as mean ± standard deviation of the three replicates for each experimental condition. Results for Is. 194, Is. 202, and Is. 42 at 7 days are missing due to accidental reasons. Identical letters indicate the lack of a significant difference in PAH degradation between different strains.

Abiotic losses after 3 and 7 days of incubation accounted for 17 and 37%, respectively, for phenanthrene, and for 3 and 7%, respectively, for anthracene, probably due to their medium-to-high volatility. Bioconversion of the three-ring compounds mainly occurred in the first 3 days of incubation in all microcosms. Phenanthrene was more susceptible to microbial attack, as its degradation was higher than 87 and 95% after 3 days and 1 week of incubation, respectively. Degradation of phenanthrene by all tested isolates with respect to the heat-killed controls after 3 days did not show significant differences (Kruskal-Wallis test: χ^2^_5_ = 8.158, *p* = 0.148). The model including strains sampled after both 3 and 7 days did not show any significant difference either (Kruskal-Wallis test: χ^2^_5_ = 10.26, *p* = 0.07; Figure 3A). On the contrary, anthracene was more recalcitrant to microbial degradation (Figure 3B). However, strains differed in anthracene degradation after 3 days of incubation (Kruskal-Wallis test: χ^2^_4_ = 10.7, *p* = 0.030). Particularly, Is.112 (*Sphingomonas* sp.) showed the overall highest anthracene degradation (58 ± 4% SE) but was significantly different only from Is.42 (*Methylobacterium* sp.) (*z* = 3.10, *p* = 0.019). After 7 days, no further significant degradation of anthracene was observed for any of the strains (Kruskal-Wallis test: χ^2^_5_ = 9.0, *p* = 0.109).

**Figure 4:**
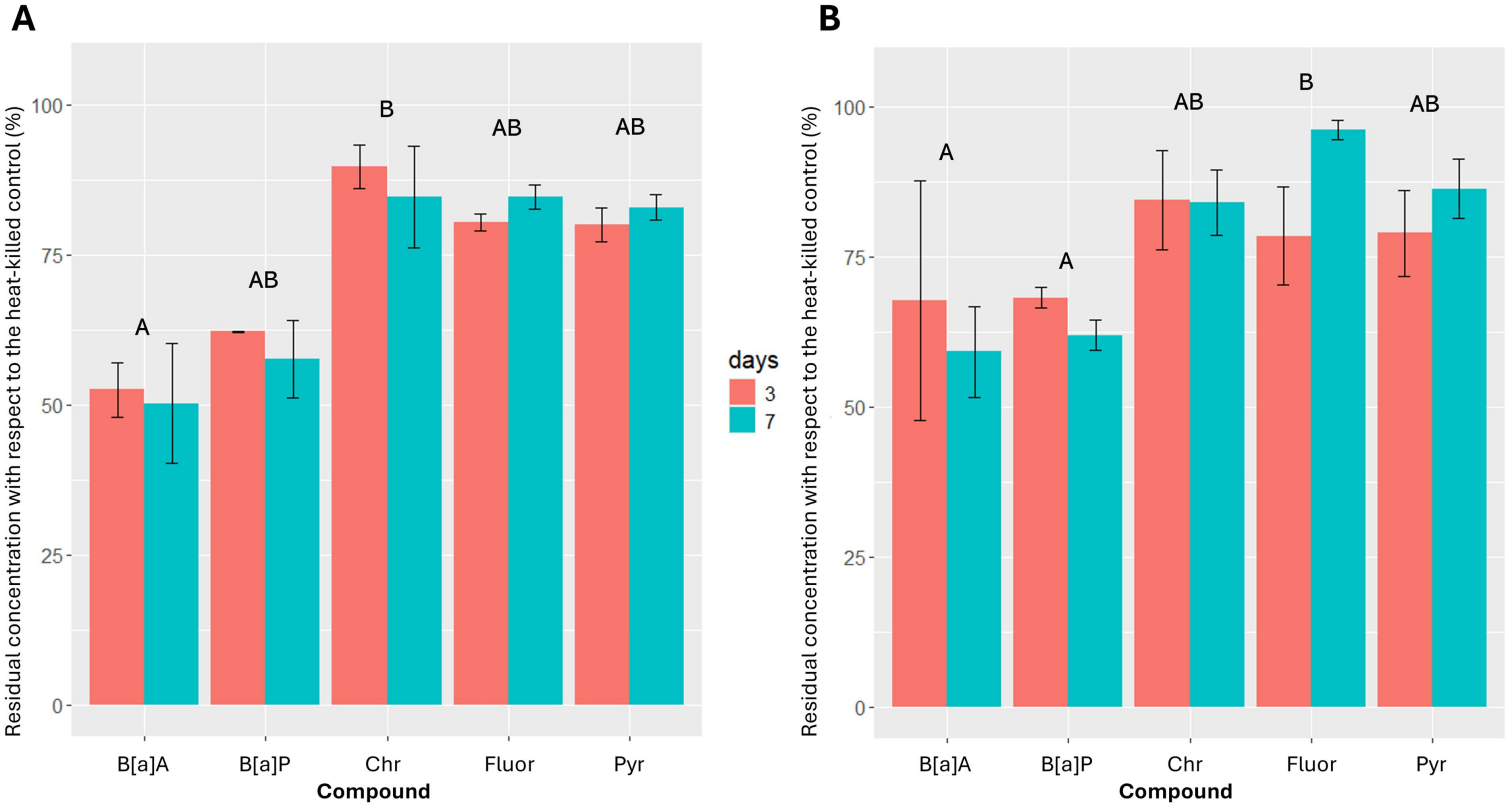
Benzo[a]anthracene (B[a]A), benzo[a]pyrene (B[a]P), chrysene (Chr), fluoranthene (Fluor) and pyrene (Pyr) residual concentrations, after 3 and 7 days of incubation at 25°C for Is. 103 (A) and Is. 112 (B). Results are presented as mean ± standard deviation of the three replicates for each experimental condition. Identical letters indicate the lack of significant differences between different compounds for the same strain.

Most of the tested strains, particularly those belonging to genus *Methylobacterium* (Is. 4, Is. 42 and Is. 194), were not able to degrade high molecular weight PAHs. Only Is. 103 and Is. 112, both belonging to genus *Sphingomonas*, were able to catabolize all the high molecular weight PAHs tested (Figure 4). As demonstrated by the statistical analysis, both isolates generally exhibited their hydrocarbonoclastic activity in the first 3 days of incubation, with no significant differences between the two strains and the two considered times (Kruskal-Wallis test: χ^2^_3_ ≤ 7.62, *p* ≥ 0.055). Overall, benzo[a]anthracene and benzo[a]pyrene removal were always higher than 32%, while both strains were less efficient in the removal of the other four-ring PAHs (4–21% for fluoranthene, 14–21% for pyrene, 10–16% for chrysene). Is. 103 degraded chrysene significantly less than benzo[a]pyrene at 3 days (*z* = 3.29, *p* = 0.010). Is. 112 degraded fluoranthene significantly less than benzo[a]anthracene and benzo[a]pyrene at 7 days (*z* ≥ 2.74, *p* ≤ 0.031).

#### 3.1.3 Plant growth promoting traits

A total of 9 isolates, both hydrocarbonoclastic strains and other phyllospheric bacteria isolated in the pre-screening stage, were chosen to be tested for their plant growth promotion traits (Table 1), including a *Rhizobium* strain. Although this is a characteristic genus of the rhizosphere, its presence in the aerial parts of plants can prove its ability to migrate in different plant compartments through the plant vascular system, as previously described (Knief et al., 2012; Müller et al., 2016; Shao et al., 2023). Table 2 reports the results of all tests.

Auxins are phytohormones produced by vegetation and associated bacteria, which mainly regulate plant growth. In the test conducted in this study, most isolates were able to produce auxins, ranging from weakly positive to outstanding (Table 2). Particularly, Is.107 (*Aeromicrobium* sp.) resulted the most efficient auxin producer, followed by Is. 63 (*Massilia* sp.), but also other strains such as Is.4 (*Methylobacterium* sp.), Is.202 (*Sphingomonas* sp.) and Is.289 (*Rhizobium* sp.) tested positive for auxin production. On the contrary, none of the tested bacterial strains was able to synthesize ACC-deaminase in the assay conditions.

As phosphorus and potassium are usually present in the form of insoluble salts in soil, the ability of plant-associated bacteria, particularly at the rhizosphere level, to solubilize these nutrients to promote their bioavailability is fundamental. Is. 289, classified as *Rhizobium* sp., showed a remarkable ability in P solubilization in both performed tests, as well as Is. 35 (*Curtobacterium* sp.), which however grew exclusively on Pikovskaya medium. Conversely, Is. 2 and Is. 4 (both *Methylobacterium* sp.) showed a weak phosphate solubilization activity only, while Is. 107 (*Aeromicrobium* sp.) tested positive, but only in one of the performed tests. The other tested strains resulted negative in both assays. In contrast, all tested strains could not solubilize K, and only Is. 2 tested positive in the siderophore production assay. Finally, most of the studied strains showed cellulase production, although at different degree, and all of them were able to synthesize cellulose.

### 3.2 Biodegradation of naphthalene on ivy leaves

#### 3.2.1 Preliminary tests on *Hedera helix* leaves

Naphthalene spiking of leaves gave consistent results. In fact, basal concentration of naphthalene on leaves before spiking was highly uneven (3.1 ± 2.5 ng cm^−2^), while after spiking naphthalene concentrations measured on leaves sampled from different parts of the plants were homogeneous (6.8 ± 0.0 and 7.4 ± 0.8 ng cm^−2^ for the two analyzed plants, respectively). The proposed method of manual spiking was therefore considered reliable for small plants such as those used in this study.

To assess the reliability of the proposed leaf sterilization method, plate counts were carried out on a sample of untreated leaves and a sample of leaves treated with a 1% NaClO solution, collected from the same plant. While the abundance of culturable bacteria was 24 CFU cm^−2^ on untreated leaves, no visible colonies were observed in plates from sterilized leaves. The treatment with 1% NaClO was therefore considered effective in reducing bacterial abundance by at least two orders of magnitude.

As shown in Table 4, all tested isolates persisted on ivy leaves even after 8 weeks from inoculation. However, *Rhizobium soli* abundance was greatly reduced over time, starting from 4 weeks after inoculation. The addition of Tween 20 to *Sphingomonas* sp. inoculum did not significantly improve persistence; on the contrary, its abundance was reduced more over time in comparison to the same inoculum without Tween 20. Therefore, the addition of this surfactant was not considered any more in further experiments. Due to its persistence and the widespread ability to live in the phyllosphere, *Sphingomonas* sp. was chosen as the inoculum for greenhouse experiments.

**Table 4:** Percentage of Illumina reads classified in the same genus of the inoculated strain.

Time (weeks)	*Curtobacterium*	*Rhizobium*	*Sphingomonas*	*Sphingomonas* + Tween20
0	98 ± 1%	96 ± 1%	96 ± 0%	89 ± 2%
1	89 ± 3%	95 ± 1%	95 ± 1%	80 ± 13%
2	94 ± 5%	91 ± 1%	98 ± 0%	78 ± 16%
4	88 ± 2%	66 ± 13%	95 ± 2%	88 ± 7%
8	91 ± 3%	65 ± 1%	97 ± 1%	94 ± 1%

As reported in Table 5, naphthalene concentrations were stable in the first 96 h after spiking. It was therefore assumed that at least short-term abiotic losses could be considered negligible on leaves. This is quite different from what observed in flasks, where abiotic losses were very high even after 24 h only. This is probably due to the very different incubation conditions: indeed, naphthalene sublimation is much more favored in flasks, especially due to shaking and to its moderate solubility in water, than on leaves, where cuticle waxes can effectively absorb and/or solubilize the molecule.

**Table 5:** Naphthalene concentrations on NaClO-treated leaves to assess abiotic losses of the compound.

Time (h)	Naphthalene concentration (ng cm^−2^)
0	10.9 ± 0.6
6	11.9 ± 0.6
24	13.6 ± 0.1
48	10.3 ± 0.2
96	11.0 ± 1.3

Concentrations are referred to one leaf side, since leaves were spiked with naphthalene on the upper side only. Each value is the average of duplicate samples, collected from two different plants as described in section 2.2.1.4. Results are reported as mean ± SE.

#### 3.2.2 Greenhouse experiments

**Figure 5:**
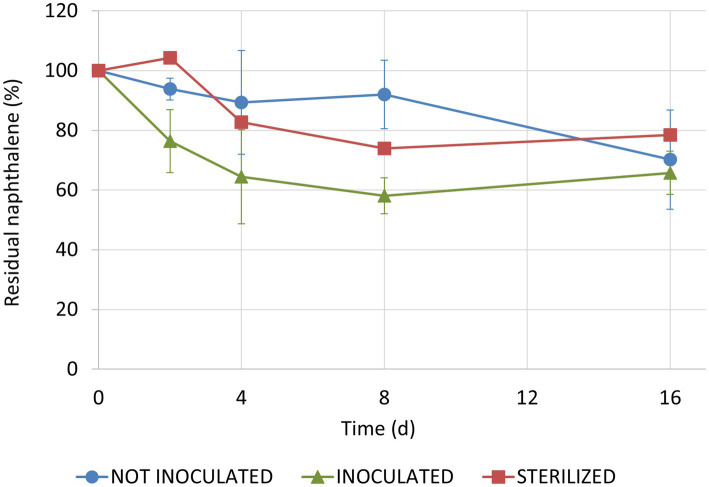
Residual naphthalene concentrations on ivy leaves in the three tested conditions. One sterilized plant was excluded from the analysis because there was evidence of rapid bacterial recolonization of leaves.

One sterilized plant was removed from the analysis because there was evidence of rapid bacterial recolonization of leaves. Therefore, we had one replicate only for this condition. Naphthalene concentration on leaves decreased over time in all tested conditions (*F*_1, 22_ = 28.80, *p* < 0.001) with no overall significant differences between inoculated and not-inoculated conditions (*t*_22_ = 1.61, *p* = 0.263; Figure 5). In fact, after 16 days, residual naphthalene accounted for 66 ± 7% and 70 ± 17%, respectively. In the sterilized condition, the percentage of residual naphthalene stopped at 80%. However, when considering the first 4 days of the experiment only, the difference between inoculated and not-inoculated conditions was significant (*t*_12_ = −2.78, *p* = 0.041). Particularly, naphthalene degradation occurred earlier on plants inoculated with *Sphingomonas* sp. In fact, half-life (*t*_1/2_) of naphthalene, calculated assuming first order kinetics for the first 4 days of the experiment, was 6 and 24 days for inoculated and non-inoculated plants, respectively.

**Figure 6:**
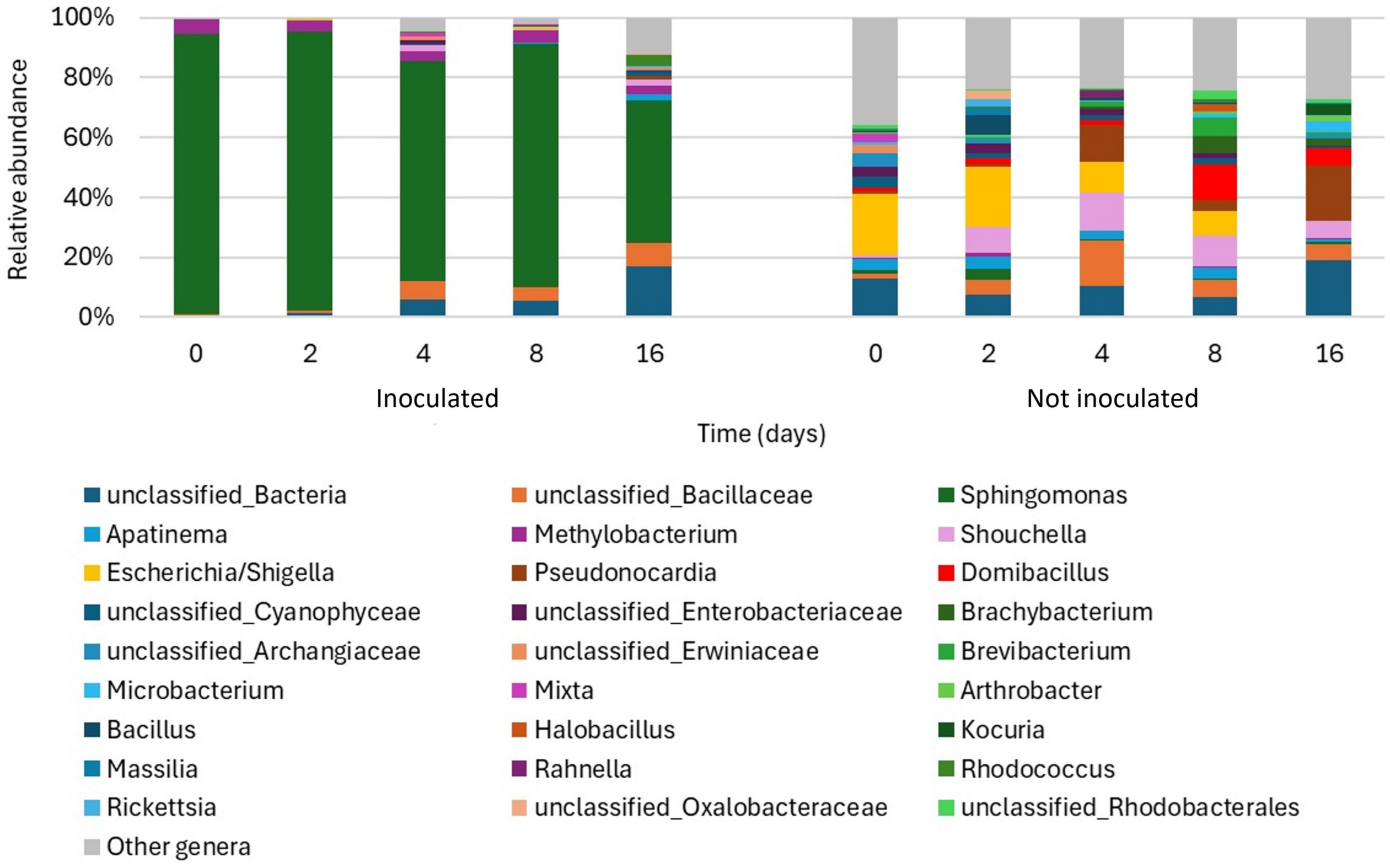
Barplots of the relative abundance of the main bacterial genera in each ivy leaf sample. All genera not reaching 2% of relative abundance in at least one sample were grouped under “Other genera”.

After removing non-bacterial ASVs, a total of 840,849 sequences were obtained, ranging between 4,557 and 160,892 per sample. As shown in Figure 6, the relative abundance of inoculated *Sphingomonas* sp. decreased over time, starting from 94% at the beginning of the experiment down to 47% after 16 days. *Methylobacterium* was consistently present during the entire duration of the experiment, while the relative abundance of unclassified members of the family Bacillaceae started increasing after 8 days. Moreover, *Rhodococcus* reached 4% of abundance after 16 days. The non-inoculated plants hosted much more heterogeneous bacterial communities. At the beginning of the experiment, members of the family Enterobacteriaceae, and especially the genus *Escherichia/Shigella*, were particularly abundant, constituting 24% of the community. However, their abundance gradually decreased over time, while the abundances of other genera consistently increased, such as *Pseudonocardia*, which reached 18% after 16 days, *Domibacillus*, which reached 12 and 6% after 8 and 16 days, respectively, and *Shouchella*, which fluctuated between 6 and 13% during the whole experiment starting from day 2. Finally, the abundance of unclassified members of the family Bacillaceae increased up to 15% after 4 days and then decreased again to 5–6%, while the genus *Bacillus* was particularly abundant only at day 2 (7%). According to the analysis of hydrocarbon catabolic potentials, both inoculated and non-inoculated leaves hosted bacterial populations that possibly have naphthalene dioxygenases, whose relative abundance increased over time (Supplementary Table S4). However, the potential ability to degrade naphthalene by populations hosted by untreated plants was higher, accounting for 2.5 and 6% at 16 days, for inoculated and non-inoculated plants, respectively. The low abundance of potential naphthalene degraders in inoculated plants is mainly due to the fact that, unfortunately, *Sphingomonas* genomes included in the dataset for functional analysis did not carry any genes coding for naphthalene dioxygenases (Khot et al., 2022). Nevertheless, although this certainly constituted a bias, it allowed us to better estimate the potential contribution of autochthonous bacteria to naphthalene degradation.

### 3.3 Microbial diversity in tree cavity organic soil

#### 3.3.1 Bacterial communities

**Figure 7:**
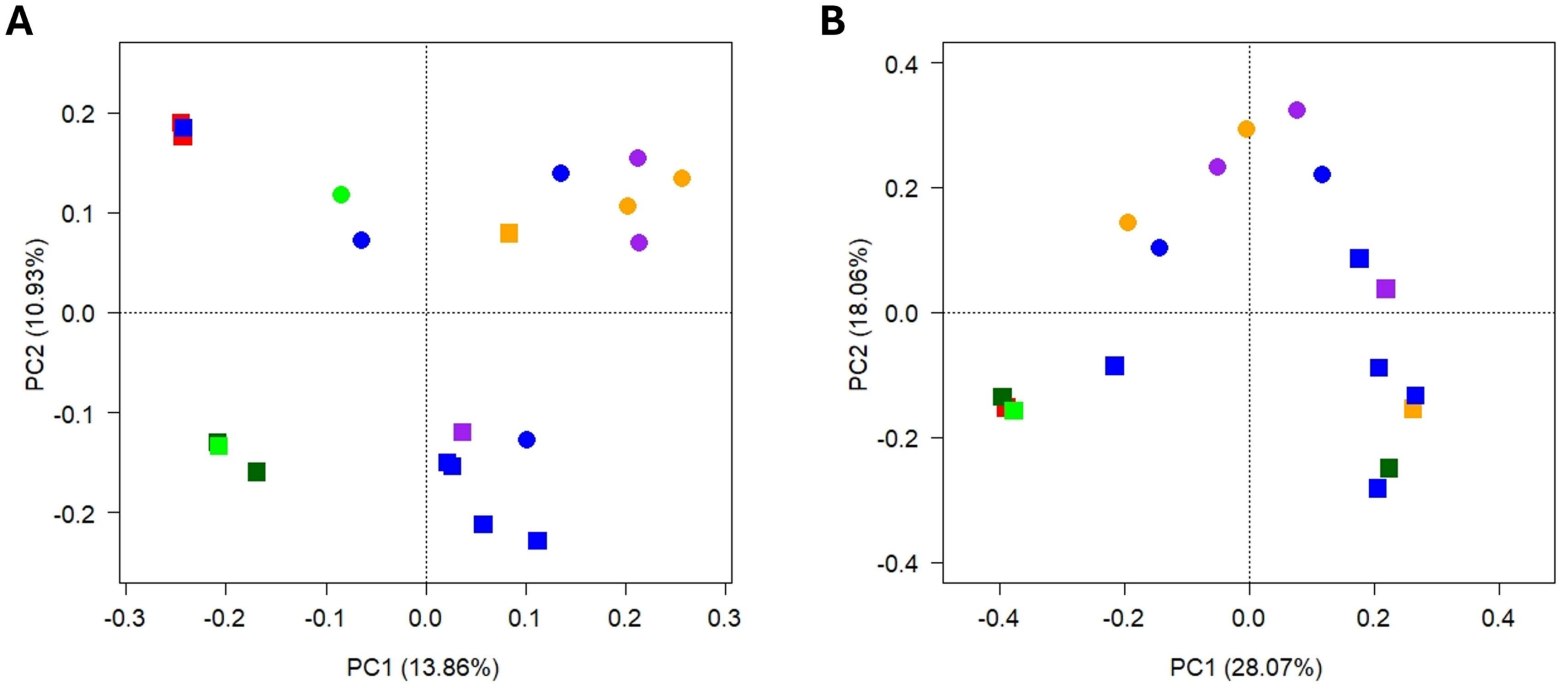
PCA biplot on Hellinger-transformed abundance of TCOS bacterial (A) and fungal (B) ASVs. Symbols denote samples according to season (shape) and tree species (color): square = autumn; dots = spring; red = cedar; orange = magnolia; blue = walnut; light green = oak; dark green = plane tree; purple = lime. The proportion of variance explained by the first two PCA axes is reported.

**Figure 8:**
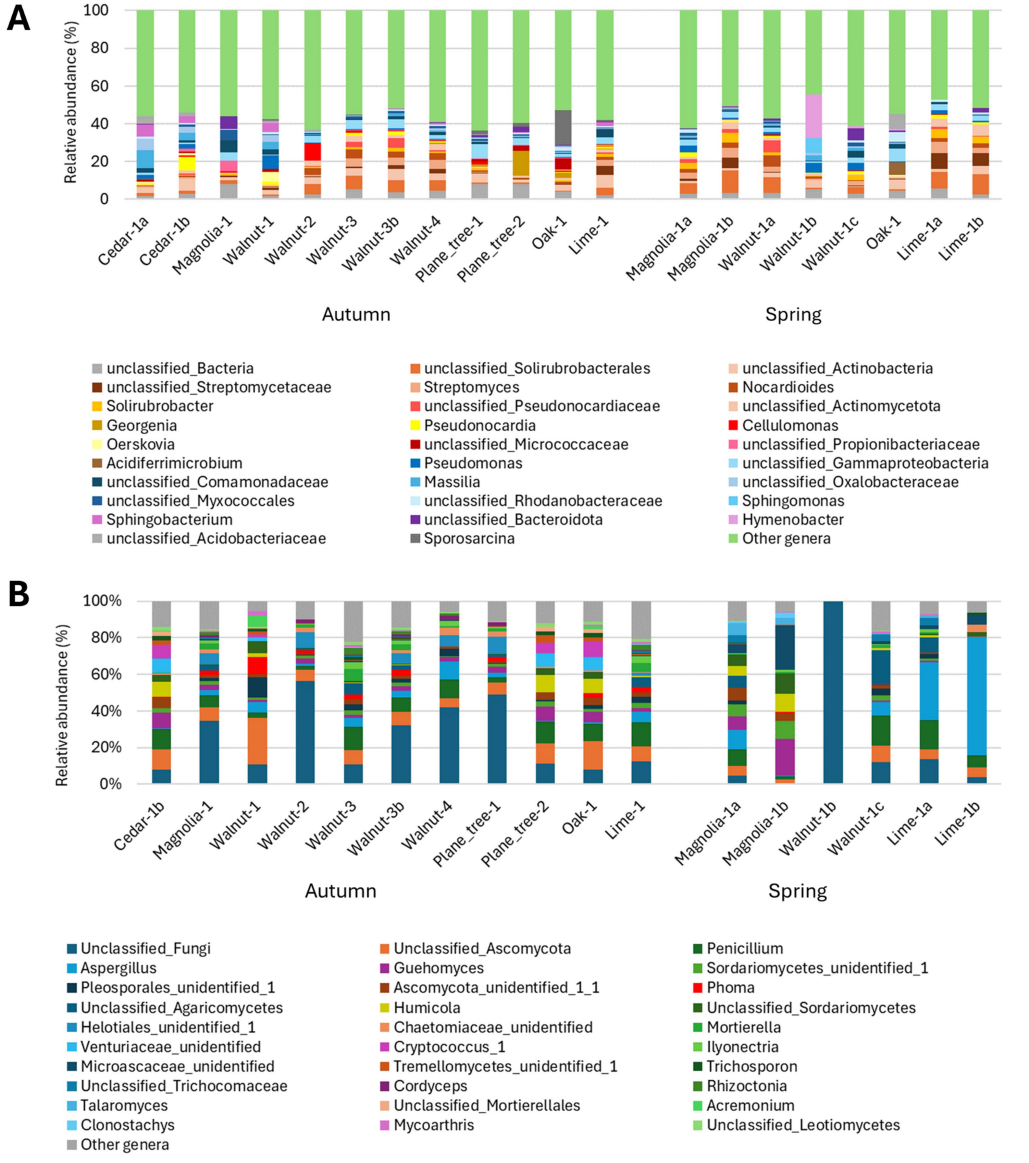
Barplots of the relative abundance of the main bacterial (A) and fungal (B) populations in each TCOS sample, classified at genus level or to the deepest taxonomic rank achievable. (A) Populations belonging to different phyla are represented with different color palettes (Actinomycetota: yellow/orange/brown; Pseudomonadota: blue; Bacteroidota: purple; other phyla and unclassified Bacteria: gray). All populations not reaching 5% of relative abundance in at least one sample were grouped under “Other genera” (color: green); this group includes 471 genera and 181 unclassified taxa at different taxonomic ranks. (B) All populations not reaching 2% of relative abundance in at least one sample were grouped under “Other genera”; this group includes 112 genera and 78 unclassified taxa at different taxonomic ranks.

After quality filtering and removal of non-bacterial ASVs, a total of 321,652 sequences were obtained, ranging between 896 and 132,723 per sample (Supplementary Table S5). PCA suggests a clustering of samples according to season and, to a lesser extent, to tree species (Figure 7A). Bacterial community structure was highly heterogeneous among different TCOS samples (Figure 8A). Members of phylum Actinomycetota clearly dominated the community in many samples, ranging 9.1–39.5%, particularly walnut samples in autumn, magnolia samples in spring, and lime samples in both seasons. At lower taxonomic ranks, however, different taxa were often unevenly distributed among samples collected from different trees and/or in different seasons. Indeed, while genera *Streptomyces* and *Nocardioides* were abundant in most walnut, lime and spring magnolia samples (up to 5.8 and 6.1%, respectively), and unclassified members of order Solirubrobacterales were abundant in most samples of both seasons (ranging 0.3–11.9%), some other genera were occasionally abundant in one or few samples only, sometimes with a seasonal trend. For instance, genus *Solirubrobacter* was generally abundant in spring samples only, except for the oak sample, ranging 0.4–5.4%, while, in contrast, *Georgenia* was completely absent from spring samples and particularly abundant in plane tree and oak samples in autumn, ranging 1.2–13.3%. Genus *Pseudonocardia* was abundant in one autumn cedar sample only (7.0%), genera *Cellulomonas* and *Oerskovia* in one autumn walnut sample each (9.1 and 5.3%, respectively), and *Acidiferrimicrobium* was abundant in the spring oak sample only (7.1%). Moreover, unclassified members of family Streptomycetaceae were particularly abundant in lime samples in both seasons, ranging 4.5–8.5%. In addition to Actinomycetota, a few members of phyla Pseudomonadota were also observed in all samples, ranging 5.2–22.6%. Among them, genera *Pseudomonas* and *Massilia* were particularly abundant in cedar and some walnut samples, ranging 2.2–7.1% and 1.8–9.3%, respectively, while *Sphingomonas* was abundant in one spring walnut sample only (8.0%). Finally, some members of other phyla were occasionally abundant in one or few samples only. For instance, within the phylum Bacteroidota, *Sphingobacterium* was abundant in one autumn walnut and in cedar samples (ranging 3.3–6.4%) and *Hymenobacter* was abundant in one spring walnut sample (23.0%). Genus *Sporosarcina*, of phylum Bacillota, was abundant in the autumn oak sample only (18.4%).

Results from the analysis of hydrocarbon catabolic potentials (Supplementary Table S6) indicated that the hydrocarbon degradation potential in aerobic conditions, although highly heterogenous among different trees and between seasons, was pronounced, especially for alkanes, including the long-chain ones, and polyaromatic hydrocarbons. In fact, the relative abundance of putative alkane-degraders, based on AlkB estimated abundance, ranged from 1.4 to 12.4%, while the potential for long-chain alkane degradation (LadA-alpha monooxygenases, range: C_15_-C_36_) was even higher, ranging from 1.9 to 16.2%. The abundance of bacterial populations possibly having naphthalene dioxygenases ranged from 0.6 to 12.4%, while the potential for aerobic degradation of monoaromatic compounds was estimated to be lower, generally in the range 0–1.7%. Finally, the potential for anaerobic degradation of alkanes and monoaromatic hydrocarbons was very low, generally close to 0% and never exceeding approximately 2%. Anaerobic degradation of polyaromatic hydrocarbons was estimated to be completely absent.

#### 3.3.2 Fungal communities

After quality filtering, a total of 402,188 sequences were obtained, ranging between 96 and 185,031 per sample (Supplementary Table S5). The three samples with less than 500 valid sequences were excluded from further analyses. PCA suggests a clear clustering of samples according to season, especially along the second axis, while they did not cluster according to tree species (Figure 7B).

Similarly to bacterial communities, also fungal communities showed high heterogeneity among TCOS samples (Figure 8B). Besides many fungal taxa that were unclassified at different taxonomic ranks, the most abundant genera were highly widespread cosmopolitan fungi, such as *Aspergillus, Penicillium* and *Cryptococcus*. Particularly, *Penicillium* was abundant in almost all samples, ranging 2.0–16.2%, while *Cryptococcus* was observed in some autumn samples only (1.5–8.6%), all collected from different tree species. In contrast, *Aspergillus* was more abundant in magnolia, walnut and lime samples in both seasons (1.2–64.9%), and clearly dominated fungal communities hosted by lime in spring (31.9 and 64.9% for the two analyzed samples). However, a few other genera, typically inhabiting soil and dead plant material, were abundant in some TCOS samples, showing different seasonal trends. Indeed, genera *Phoma* and *Mortierella* were abundant in some autumn samples (ranging 1.9–9.5% and 1.0–4.8%, respectively), but were almost absent from spring samples. In contrast, *Talaromyces* was particularly abundant in spring magnolia samples only (3.7 and 6.7% in the two analyzed samples). Genera *Guehomyces* and *Humicola* were observed in autumn samples collected from different tree species as well as in spring magnolia samples, with abundances up to 20.1 and 9.7%, respectively, while *Acremonium* was abundant in one autumn walnut sample only (6.4%).

## 4 Discussion

One of the most challenging aspects of ecosystem service assessment is the need to correctly identify and quantify contributions provided by the different ecosystem components. This is certainly true when targeting the urban green; indeed, trees can be considered complex systems, formed not only by the plant itself but also by several different compartments and micro-habitats, and all their associated micro- and macroscopic biodiversity. For instance, each tree micro-habitat is likely to host a peculiar microbiome, both from a taxonomic and a functional point of view. Ideally, the contribution of every tree microbiome to ecosystem services should be separately assessed, especially when considering those hosted by TreMs that are still poorly studied. Nevertheless, accurately estimating microbial activities is particularly difficult, since, at present, taxonomy-based surveys are much more common than functional studies (Singer et al., 2017). So far, attempts to quantitatively evaluate the relevance of biodegradation of airborne organic pollutants by tree microbiomes have been made almost exclusively by lab-scale experimental approaches. A first attempt to calculate the extent of naphthalene biodegradation by phyllospheric bacteria on magnolia leaves concluded that absorption onto leaves and biodegradation processes could be considered equally relevant in the removal of naphthalene from the urban air (Franzetti et al., 2020). However, significant improvements in quantification methods are still needed.

In this work, we tried to integrate information given by different techniques and at different scales (isolation and characterization of culturable strains, greenhouse experiments, taxonomic surveys of unknown microbiomes), suggesting that future research should aim at integrating culture-based, taxonomic and functional surveys, to capture a more comprehensive picture of tree-associated microbial diversity and functions. This holistic approach will be essential for quantifying ecosystem services provided by the bacteria-plant systems and optimizing the use of plant-associated microbes in addressing environmental challenges related to air pollution.

### 4.1 Diversity of tree microbiomes

Although often limited by the impossibility of correctly elucidating microbial functions, taxonomy-based surveys still constitute the first approach to investigate and characterize microbial communities, especially when available information is scarce. The present work represents one of the very first surveys characterizing bacterial and fungal communities hosted by TCOS samples in an urban area. Thoroughly characterizing these environments is the first step toward understanding their functioning, and gaining a deeper knowledge of the microbiomes hosted by TCOS will be essential for better insights into tree ecosystem services, particularly in relation to urban pollution mitigation. Our results indicated that, in TCOS bacterial communities, members of phylum Actinomycetota were clearly dominant, although a few members of phyla Bacteroidota and Pseudomonadota were also abundant in some samples. This is partially different from what reported by Dangerfield and colleagues, who, besides Actinobacteria, indicated as abundant in their canopy soil communities also members of phyla Acidobacteria, Bacteroidetes, and Verrucomicrobia, and of classes Alpha-, Beta- and Gammaproteobacteria (Dangerfield et al., 2017). Dangerfield and colleagues also observed that, at least at the highest taxonomic levels, samples from canopy and forest floor soils were generally similar, but not identical, since they mainly hosted many bacterial groups commonly retrieved in previously studied soils. Indeed, a high abundance of Actinomycetota is typical of soils (Madigan et al., 2020). Moreover, in fungal communities, we detected a few abundant genera, such as *Guehomyces, Humicola, Phoma, Mortierella, Talaromyces*, and *Acremonium*, which typically inhabit soil and dead plant material (Frąc et al., 2018; Glushakova et al., 2016; Wang et al., 2019). Tree cavities may form from micro-pedogenesis of epiphytic mosses, lichens or algae and necrosed bark, or from pedogenesis of debris and litter fallen from the crowns to limbs and forks or after mechanical damages, which create openings in the tree protective bark and cambium layer, allowing pathogens to enter and initiate decay processes that can lead to cavity formation (Larrieu et al., 2018). This occurs frequently in urban areas, due to both natural and human-induced injuries; cavities are then filled with organic soil deriving from various organic debris (leaves, twigs, dead organisms and droppings). Both the origin of these cavities and the ongoing accumulation of organic material that is eventually degraded to form tree cavity soil could influence the communities that inhabit them. In fact, leaves, pieces of branches, or bark that fall into these cavities carry with them a microbial community that can colonize these niches, as well as organisms that visit, inhabit, or perish within them. A careful assessment of microbiomes hosted by TCOS is therefore useful to understand and reconstruct the processes leading to cavity soil formation. Our results showed that, at least in some samples, a few bacterial genera commonly retrieved in the phyllosphere were occasionally abundant, such as *Hymenobacter* and *Sphingobacterium*, (phylum Bacteroidota), *Massilia* and *Sphingomonas* (phylum Pseudomonadota) (Bulgarelli et al., 2013; Franzetti et al., 2020; Gandolfi et al., 2017; Vorholt, 2012). In contrast, fungal populations observed in TCOS were generally different from those forming phyllosphere communities (Vacher et al., 2016). This suggests that, despite pedogenetic processes altering the chemical and physical characteristics of this substrate, a few bacterial populations, likely originating from plant debris, can persist in tree cavity organic soil, at least occasionally. Further studies will be required to elucidate whether such phyllosphere-derived populations are only temporarily, and stochastically, present in cavity soil communities or they are rather ecologically relevant members of these communities. However, the clear clustering observed between samples collected in different seasons, both for bacteria and for fungi, suggests that seasonal fluctuations do generally occur in cavity soil microbial communities. Furthermore, the less relevant but still discernible difference observed among tree species, at least for bacterial communities, suggests a contribution from microbial populations hosted by plant material. Taken together, these results could indicate that the observed seasonal changes may be due to differences in inputs of parental material, in addition to those in physical and chemical environmental conditions associated with the canopy. Therefore, as already observed by Dangerfield and colleagues, TCOS appears to be a distinct substrate, whose microbiome is determined partly by the host tree and partly by canopy environmental conditions (Dangerfield et al., 2017).

In contrast, experiments conducted in controlled conditions, including manipulation of microbiomes, can easily take advantage of the extensive use of taxonomic-based surveys, which nowadays allow a relatively simple, fast and low-cost monitoring of community development. Our results from greenhouse degradation experiments demonstrated that the relative abundance of inoculated *Sphingomonas* sp. decreased over time, while the relative abundance of some gram-positive populations, belonging to the family Bacillaceae and to the *Rhodococcus* genus, increased on inoculated plants. Consistently, on untreated plants, a few gram-positive genera increased their abundance, such as *Pseudonocardia, Domibacillus, Shouchella*, as well as some unclassified members of the family Bacillaceae. The genus *Pseudonocardia* is well known for its potential degradation capabilities toward hydrocarbons, especially toward PAHs; bacteria belonging to this genus have been previously isolated from contaminated soils (Bao et al., 2022; Chen et al., 2018). Actinomycetota, to which the genus *Pseudonocardia* belongs, are generally characterized by slower growth kinetics, which could explain the increase in its abundance only after 16 days on inoculated plants. The family Bacillaceae also includes bacteria with potential degradation capabilities; in fact, this family is among the main groups of microorganisms able to aerobically degrade aromatic compounds (Basim et al., 2020; Kotoky et al., 2022; Lu et al., 2011). Furthermore, Bacillaceae have often been reported among the main culturable endophytic microorganisms at contaminated sites (Feng et al., 2017). The genera *Domibacillus* and *Shouchella* are also members of this family. Overall, the increase in the relative abundance of these populations over time can suggest a selection process on leaves due to the presence of naphthalene, which supports the growth of those bacteria capable of exploiting it as a carbon source. Results from hydrocarbon catabolic potential analysis further supported this hypothesis, since the estimated fraction of bacterial populations possibly having naphthalene dioxygenases increased over time both in inoculated and in non-inoculated plants.

### 4.2 Hydrocarbon degradation potential

Overall, our case studies demonstrated that tree-associated microbiomes likely possess several potential capabilities for mitigating air pollution.

The phyllospheric isolated strains we tested exhibited a widespread ability to degrade low-molecular-weight PAHs, particularly naphthalene and phenanthrene, which are among the most abundant PAHs that can accumulate on leaves in urban areas (Franzetti et al., 2020). Most tested strains, however, particularly those belonging to genus *Methylobacterium* (Is. 4, 42 and 194), were not able to degrade high-molecular-weight PAHs. This result is consistent with the literature, as these prokaryotes are principally described as facultative methylotrophs, usually growing at the expense of single or short-chain carbon compounds (Zhang et al., 2021). In fact, one of the main traits of the bacteria belonging to this genus is their ability to grow on the methanol released by plants, and for this reason they are common inhabitants of the phyllosphere (Knief et al., 2010; Rani et al., 2021). Nevertheless, bacteria belonging to genus *Methylobacterium* were sometimes retrieved in historically contaminated sites (Chikere et al., 2020; Ventorino et al., 2014), and they have been also described as able to catabolize different monoaromatics and heterocyclic xenobiotic compounds, such as explosives like trinitrotoluene (TNT), hexahydro-1,3,5-trinitro-1,3,5-triazene (Van Aken et al., 2004), dimethyl isophthalate (Li and Gu, 2007) and methyl tert-butyl ether (MTBE) (Lalevic et al., 2012). On the contrary, Is. 103 and Is. 112, both belonging to genus *Sphingomonas*, were able to catabolize all the high-molecular-weight PAHs tested, although with different efficiency. Particularly, both benzo[*a*]anthracene and benzo[*a*]pyrene removal were above 32%, while the two isolates were not equally efficient in the removal of the other tetra-rings such as fluoranthene, pyrene and chrysene. *Sphingomonas* spp. are well known for their ability to catabolize aromatic compounds (Asaf et al., 2020) and, more specifically, PAHs (Asaf et al., 2020; Peng et al., 2008). It is particularly noteworthy that these strains were isolated as endophytes. Although endophytic bacterial strains are mostly known for their plant-growth promoting properties (Cui et al., 2024), it has been hypothesized that they might also play a role in hydrocarbon degradation (Weyens et al., 2015). Indeed, since airborne lipophilic molecules, such as VOCs and PAHs, are known to be absorbed onto the wax layer of leaf cuticle, and then enter the leaves of many plants (Terzaghi et al., 2021; Weyens et al., 2015), endophytes can easily come in contact with pollutants and possibly detoxify a part of them through degradation, transformation or sequestration (Weyens et al., 2015).

Results from greenhouse degradation experiments demonstrated that naphthalene degradation occurred on both inoculated and non-inoculated leaves. A faster decrease in contaminant concentration on inoculated leaves, as indicated by the lower half-life compared to non-inoculated leaves, was observed in the first few days of the experiment only, and was probably due to a high activity by inoculated *Sphingomonas*. However, when considering later times, there were no differences between the inoculated and non-inoculated plants. Overall, the obtained results suggest that two concomitant phenomena occurred in the second part of the experiment: the disappearance of the inoculum from inoculated plants, and the enrichment, in communities hosted by non-inoculated plants, of potential degrading bacteria that were already present on leaves.

Both isolated strains and phyllospheric communities on ivy leaves degraded PAHs, and particularly naphthalene, in a short time, ranging from 24 h to a few days. Ivy autochthonous microbiomes, which were not previously adapted to use naphthalene as carbon source, were the slower ones, and apparently started degrading the pollutant only after 16 days. However, considering greenhouse experiments as the most suitable to resemble field conditions, we may infer a potential for moderately rapid adaptations of tree microbiomes to changing atmospheric concentrations of pollutants, e.g., due to seasonality. Results from catabolic potential analysis, both on ivy phyllosphere and on TCOS, further supported the hypothesis of a widespread hydrocarbon degradation potential in tree microbiomes. Although certainly biased, due to the scarcity or absence of available genomes for many understudied bacterial genera, and to the impossibility of assessing a degradation potential for unclassified populations, this taxonomy-based functional analysis can provide a rough estimate of the hydrocarbon-degrading abilities of bacterial communities in tree compartments (Khot et al., 2022). Notably, it indicated that TCOS microbiomes have a significant potential for aerobic degradation of alkanes, including the long-chain ones, and polyaromatic hydrocarbons. Particularly, the degradation potential toward polyaromatic hydrocarbons, reaching values up to 8.0% for spring samples and up to 12.4% for autumn samples, was estimated to be higher than that developed on non-inoculated ivy leaves during the greenhouse experiment (up to 6.5%). This is consistent with the hypothesis that TCOS microbiomes are constantly exposed to a range of airborne and soilborne pollutants, which may possibly accumulate in cavities. Future research should aim at elucidating the extent of this process and its relative importance in exerting selection processes on microbial populations.

### 4.3 PGP properties of isolates

Besides hydrocarbonoclastic abilities, PGP properties are particularly valuable, since bacteria possessing one or more of them can positively affect plants in a number of different ways, resulting, for example, in: increased plant biomass or increased root and shoot length; increased nitrogen, phosphorus or iron content; enhanced seed germination; increased disease resistance and tolerance to environmental stresses (Benitez-Alfonso et al., 2023; García-Fraile et al., 2015).

Most isolates resulted positive for auxin production. Kuffner and collaborators observed the production of auxins, in particular IAA, in bacteria belonging to genera *Rhizobium* and *Sphingomonas* associated with *Salix caprea* grown in a site characterized by heavy metal contamination (Kuffner et al., 2010). This is particularly interesting, as the urban setting from which *Q. ilex* leaves were sampled is also characterized by the presence of heavy metals both in PM10 and at ground level (https://www.arpa.umbria.it/monitoraggi/aria/metallideposizioni.aspx). Moreover, vegetation responds to stressful conditions by secreting ethylene, a hormone that inhibits root elongation and nodulation, promotes senescence, and even prevents auxin transport. Bacteria associated with plants can synthesize an enzyme able to cleave the precursor of this hormone, 1-aminocyclopropane-1-carboxylic acid (ACC), called ACC deaminase (Glick et al., 2007; Shahid et al., 2023), thus helping the plant cope with environmental stresses. However, none of the tested bacterial strains was able to synthesize ACC-deaminase. Metal solubilization and metal uptake traits are usually more common among rhizospheric bacteria (Seneviratne et al., 2017); therefore, the negative results we obtained for most isolated strains were not surprising. Finally, among PGP traits, characteristics such as vegetable macropolymer synthesis and degradation are particularly relevant to establish plant-bacteria relationships. For example, cellulase production allows potential endophytes to enter the plant tissues, colonize them and then migrate to different plant districts (Hung et al., 2007; Papik et al., 2020). Most of the studied strains were positive both in the cellulase production and in the cellulose biosynthesis assays. This polymer is especially relevant to the bacterium-host association as it is a component of the extracellular polymeric substance (EPS) needed for the development of microbial biofilms on plant surfaces (Abidi et al., 2022; Robledo et al., 2012; Serra et al., 2013). Particularly, the production of lytic enzymes could have a role in the protection of plants from diseases caused by phytopathogen growth and spread. Indeed, bacteria secreting cellulases have been often reported as involved in biocontrol processes against fungal pathogens as these enzymes are able to hydrolyse fungal structural components. For instance, Ashwini and Srividya observed the mycolytic activity of cellulase and glucanase produced by *Bacillus subtilis* BC2 toward *Colletotrichum gloeosporioides* OGC1, responsible for anthracnose disease in different types of plants (Ashwini and Srividya, 2014). The endophytic strain *Bacillus pumilus* JK-SX001 was demonstrated able to promote poplar growth in greenhouse experiments through the secretion of lytic enzymes such as cellulase and protease, through growth suppression of the phytopathogens that cause poplar canker (Ren et al., 2013). In contrast, there is also evidence that the addition of cellulase to a plant-fungus system enhanced *Colletotrichum coccodes* virulence toward *Abutilon theophrasti* due to wall loosening mechanisms operated by the hydrolytic activity of the enzyme (Babalola, 2007).

Overall, our isolates mostly possessed some PGP properties, such as auxin production, cellulase production and cellulose biosynthesis, which are particularly relevant to establish plant-bacteria relationships (García-Fraile et al., 2015; Robledo et al., 2012). When considering these data together with their widespread abilities to degrade low-molecular-weight PAHs, we can hypothesize a two-fold contribution to plant-bacteria systems, where microbiomes are responsible both for direct degradation of air pollutants and for sustaining the plant metabolism and its resistance to environmental stresses.

### 4.4 Concluding remarks

Overall, the diversity of micro-habitats and conditions studied in this work emphasizes the multifaceted role of urban trees as hosts for complex microbiomes, which are notably different when separately considering the various tree compartments. This is particularly relevant for urban green planning and management aspects. Indeed, urban green management often lacks an ecological perspective, focusing mainly on risk factors rather than the importance of tree components for biodiversity and ecosystem services. This imbalance can reduce the effectiveness of these services. For example, one of the most widespread practices in urban environments is topping, which generally involves a drastic reduction of the canopy. This leads to a late formation of leaves, especially if it occurs when buds have already sprouted, which can even reach 30/35 days. This in turn affects the development of phyllospheric microbiomes and ecosystem services for over a month. Topped trees also produce fewer but denser leaves, with altered physiological properties that can lead to premature leaf loss and reduced ecosystem services. Managing larger, older trees poses additional challenges, as their risk of breaking or falling leads to extensive pruning or removal. Structural irregularities like cavities and forks are often seen as flaws, resulting in repeated interventions that reduce TreMs and their associated materials, including TCOS, in turn causing an impairment of ecosystem services provided by these peculiar ecosystems. While ensuring that risk reduction is maintained, specific actions should be taken to conserve trees and these increasingly rare structures, both by applying solutions that already exist, and by developing new ones, such as braces stabilization cables (Rust, 2015). Therefore, the study of tree microbiomes not only enhances our comprehension of tree-related ecosystem services but also underscores the importance of preserving trees, especially those hosting complex structures, which generally are the largest and oldest ones as already suggested by other authors (Lindenmayer, 2017; Manning et al., 2006).

In this work, tree-associated microbiomes, whether on leaves or within tree cavity soils, demonstrated a consistent ability to degrade hydrocarbons, especially the polyaromatic ones, across various conditions and tree species, thus contributing to the overall tree-driven contribution to airborne hydrocarbon biodegradation. Each micro-habitat provides a unique environment for the hosted microbiomes, yet they all share the functional capacity to degrade pollutants. These results reinforce the hypothesis that these microbial communities are crucial for ecosystem processes and must therefore be included in ecosystem services assessment.

Future research should focus on better characterizing the microbial metabolic pathways involved in pollutant degradation and exploring the potential for harnessing these communities in bioremediation efforts. This foundational knowledge will be the first step toward developing innovative, microbe-driven sustainable solutions. Studies on artificially manipulated systems in controlled environments, such as greenhouses or closed chambers, can give valuable information on the ability of bacteria-plant systems to cope with hydrocarbon pollution. On the one hand, such artificially built plant-bacteria systems could be used to significantly increase degradation rates of polluting compounds present in the atmosphere, although they still need further development and optimization, e.g., by evaluating other possible strategies to improve the inoculum activity and degradation effectiveness and efficiency. On the other hand, studies in controlled conditions may allow a better assessment of degradation processes, especially from a quantitative point of view.

Such insights are also particularly valuable for supporting urban green planning and informing effective management policies. By integrating the ecological benefits of urban trees and their microbiomes into city planning, we can enhance the resilience and sustainability of urban environments, ultimately improving public health and quality of life.

## Data Availability

The datasets generated for this study, including raw DNA sequences, can be found in Zenodo open repository (https://zenodo.org/records/12795680) with doi: 10.5281/zenodo.12795680. Raw sequences were also submitted to Sequence Read Archive (SRA-NCBI), with accession numbers SAMN43826549, SAMN43826648, BioProject PRJNA1162784.
