# Supplementary material for: Microbiomes of urban trees: unveiling contributions to atmospheric pollution mitigation
Source: Front Microbiol. 2024 Nov 11;15:1470376. doi: 10.3389/fmicb.2024.1470376 (PMC11586189; doi:10.3389/fmicb.2024.1470376)
Supplement: Supplementary file 1 [file Data_Sheet_1.PDF]

Supplementary Material

1 Supplementary Figures and Tables

1.1 Supplementary Figures

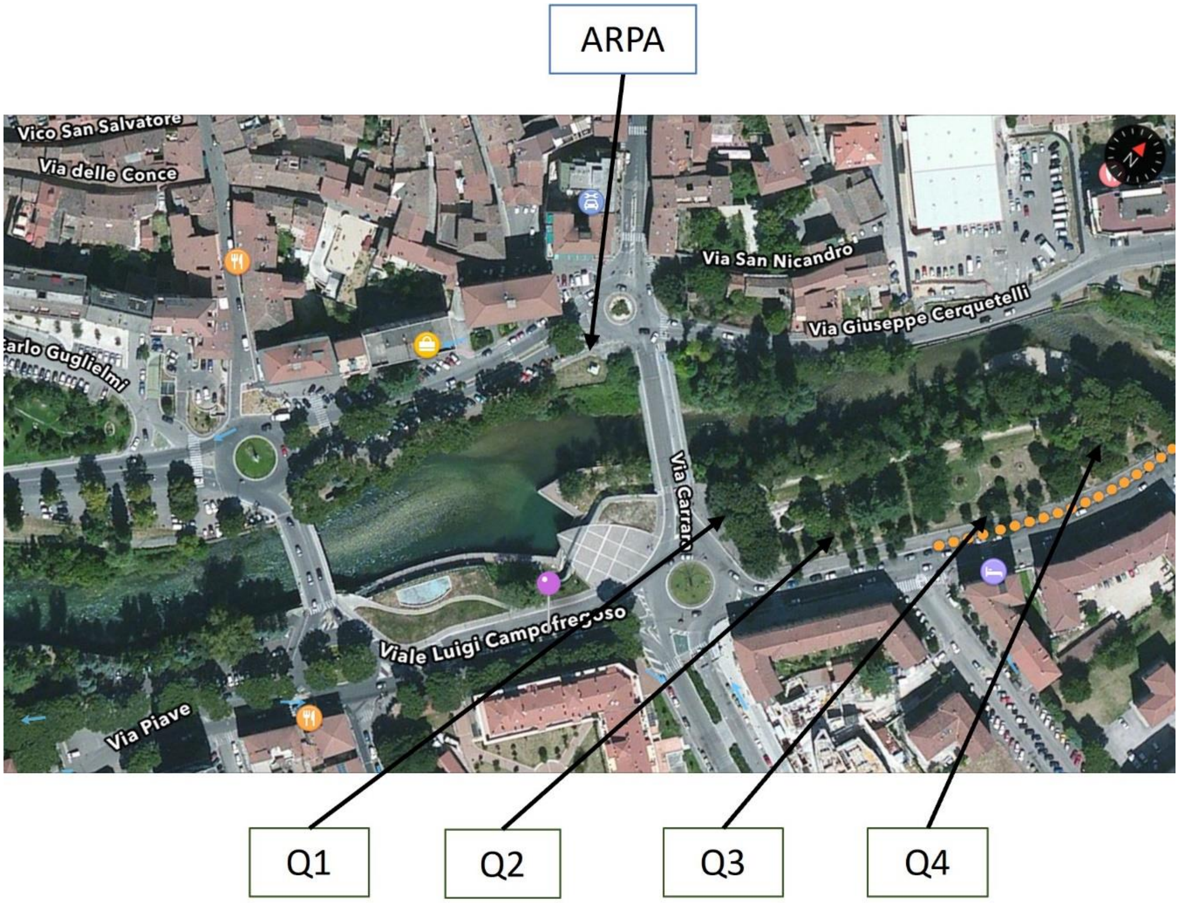

Supplementary Figure 1. Area in Terni (Italy) where *Quercus ilex* leaves were sampled to isolate phyllospheric strains (section 2.1.1). The ARPA (Regional Agency for Environmental Protection) air quality cabin (ARPA) and the four *Q. ilex* trees (Q1-Q4) are indicated on the map. Courtesy of Dr. Ermanno Federici, University of Perugia.

Supplementary Material

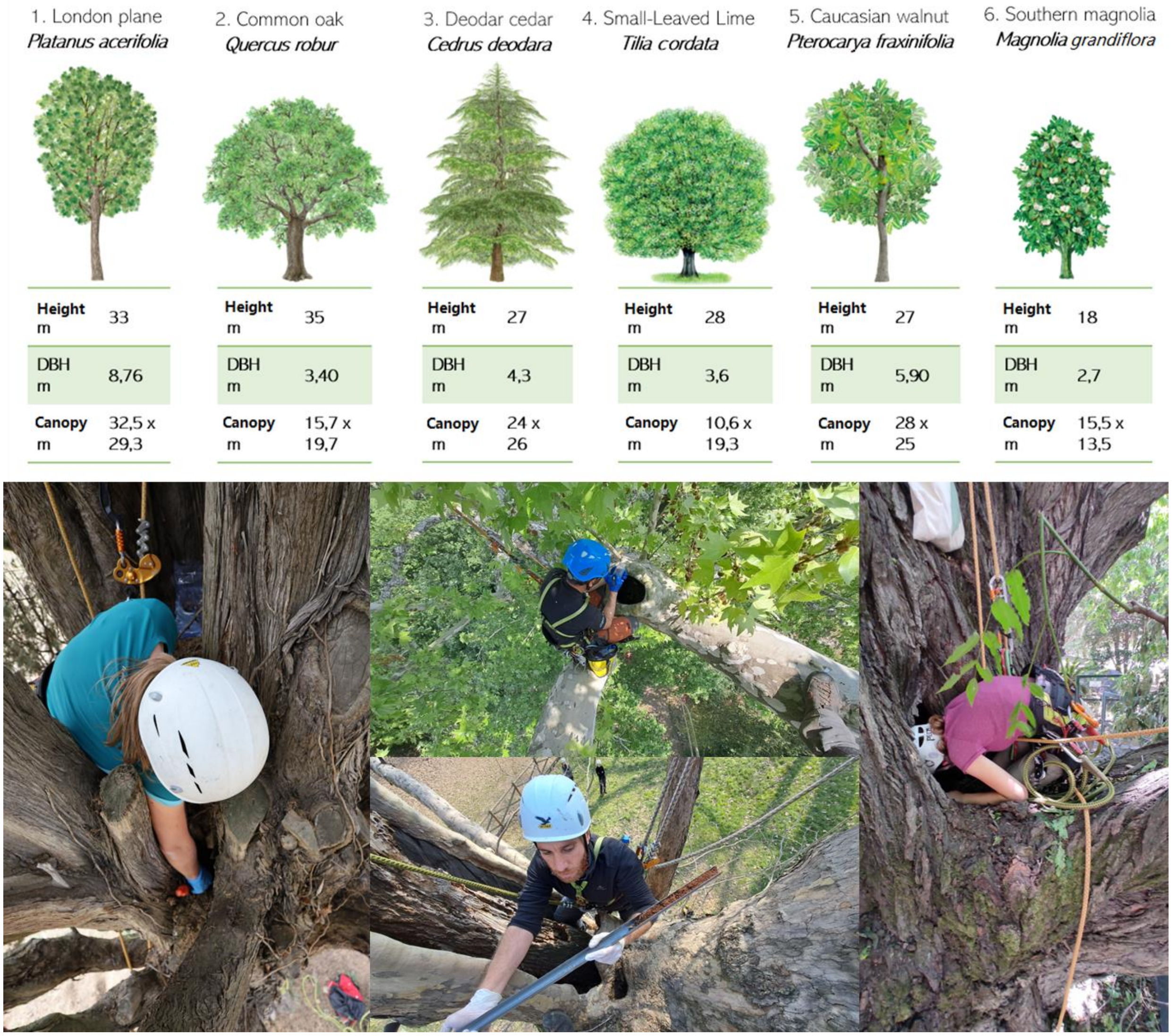

Supplementary Figure 2. Sampling of tree cavity organic soil (TCOS) in Giardini Indro Montanelli, Milan, Italy (section 2.3.1). Upper panel: diagram of sampled trees and their main characteristics. Lower panel: TCOS sampling from tree cavities.

1.2 Supplementary Tables

Supplementary Table 1. Composition of modified minimal medium Bushnell-Haas (BH) Agar, supplemented with thiamine and oligo-elements.

Components		Concentration (g L^{-1})	
Magnesium sulphate		0.20	
Calcium chloride		0.02	
Potassium dihydrogen phosphate		1.00	
Potassium hydrogen phosphate		1.00	
Ammonium nitrate		1.00	
Iron (III) chloride		0.05	
Thiamine hydrochloride		0.00001	
Oligo-element solution containing (mg mL^{-1}):		10 mL	
	$MnCl_2 \cdot 4H_2O$		4.3
	$CuSO_4 \cdot 5H_2O$		5
	$ZnSO_4 \cdot 7H_2O$		108
	H_3BO_3		145
	$FeSO_4 \cdot H_2O$		125
	$CoCl_2 \cdot 6H_2O$		59
	Na_2MoO_4		125
Agar		15	

Supplementary Table 2. List of conditions set up to evaluate bacterial ACC deaminase production.

Test	Variables
P1	With N, without glucose, with ACC
P2	Without N, without glucose, with ACC
P3	Without N, with glucose, with ACC
P4	With N, with glucose, with ACC
P5	Without N, without glucose, without ACC
P6	With N, with glucose, without ACC
P7	Without N, with glucose, without ACC

Supplementary Material

Supplementary Table 3. Isolates from *Q. ilex* phyllosphere able to use naphthalene and/or phenanthrene as sole carbon and energy source on plates (pre-screening phase). Strains in bold are also mentioned in the main text (Table 1) since they were further characterized.

Isolate n.	Isolate Code	Closest type strain *	Growth evaluation †	
			Naphthalene	Phenanthrene
**002**	**TRW_QO25P_02**	***Methylobacterium adhaesivum***	**	-
**004**	**TRW_QO25P_04**	***Methylobacterium goesingense***	***	****
005	TRW_QO25P_05	*Sphingomonas aerolata*	**	***
011	TRW_QO25P_11	*Sphingomonas hankookensis*	*	*
018	TRW_QO25P_18	*Bacillus idriensis*	****	****
**030**	**TRW_QO25P_30**	***Curtobacterium flaccumfaciens***	*	*
**035**	**TRW_QO25P_35**	***Curtobacterium oceanosedimentum***	*	*
**042**	**TRW_QO25R_01**	***Methylobacterium goesingense***	***	****
047	TRW_QO25R_06	*Rathayibacter festucae*	*	-
049	TRW_QO25R_08	*Sphingomonas aquatilis*	***	*
050	TRW_QO25R_09	*Sphingomonas cynarae*	***	*
053	TRW_QO25R_12	*Methylobacterium adhaesivum*	**	**
**063**	**TRW_QO25R_22**	***Massilia aurea***	*	*
067	TRW_QO25R_26	*Bacillus idriensis*	***	***
078	TRW_QO25K_09	N.I.	***	****
083	TRW_QO25K_14	*Microbacterium aurum*	*	-
**103**	**TRW_QI25R_01**	***Sphingomonas yunnanensis***	****	****
104	TRW_QI25R_02	*Variovorax boronicumulans*	***	***
111	TRW_QI25R_09	*Curtobacterium oceanosedimentum*	**	*
**112**	**TRW_QI25R_10**	***Sphingomonas endophytica***	****	****
114	TRW_QI25R_12	*Kineococcus radiotolerans*	*	***
135	TRW_QI25K_19	*Sphingomonas yunnanensis*	***	***
153	TRW_QI25P_17	*Kineococcus radiotolerans*	-	***
174	TRW_QI25M_07	*Rathayibacter festucae*	**	*
184	TRW_QO25M_05	*Sphingomonas aerolata*	***	***
**194**	**TRW_QO25M_10**	***Methylobacterium marchantiae***	***	****
200	TRW_QO25M_13	*Massilia aurea*	*	-
**202**	**TRW_QO25M_14**	***Sphingomonas panni***	****	****
237	TRW_QO10R_12	*Frigoribacterium faeni*	**	*
243	TRW_QO10R_18	*Sphingomonas aerolata*	****	****
257	TRW_QO10P_07	*Sphingomonas aerolata*	***	***
277	TRW_QO10P_27	*Sphingomonas faeni*	***	***

287	TRW_QI10R_06	*Variovorax guangxiensis*	**	**
288	TRW_QI10R_07	*Sphingomonas faeni*	***	***
291	TRW_QI10R_10	*Bacillus idriensis*	****	****
292	TRW_QI10R_11	*Sphingomonas aerolata*	***	***
313	TRW_QI10P_03	*Rathayibacter tritici*	*	***
343	TRW_QO10P_36	*Rathayibacter tritici*	*	-
368	TRW_QO25P_48	N.I.	*	****

* The identification is based on the best-hit against 16 rRNA sequences of type strains (NCBI database); N.I. = not identified.

† Results were read after 3 weeks of plate incubation at 25°C, by comparing biomass growth of isolates in the presence and in the absence (negative controls) of naphthalene (5 mg/plate) or phenanthrene (5 mg/plate). They were qualitatively expressed according to the following scale: - no growth; * moderate growth; ** moderate growth and colour change due to pigmentation; *** abundant growth; **** excellent growth with mucous layer development.

Supplementary Table 4. Relative abundance of bacterial populations possessing naphthalene dioxygenases, hosted by ivy leaves at different experimental times (IN = inoculated leaves; NO-IN = not inoculated leaves). The reported percentage is the sum of NdoB and non-NdoB-type naphthalene dioxygenases (see Khot et al., 2022).

**Sample**	**Relative abundance of bacterial populations hosting naphthalene dioxygenases (%)**
IN 0 days	1.4
IN 2 days	1.1
IN 4 days	1.2
IN 8 days	1.3
IN 16 days	2.5
NO-IN 0 days	4.0
NO-IN 2 days	2.7
NO-IN 4 days	4.6
NO-IN 8 days	6.5
NO-IN 16 days	6.2

Supplementary Table 5. Number of valid bacterial and fungal sequences obtained per sample in tree cavity organic soil (TCOS). Samples indicated with an asterisk (*) were discarded from subsequent analyses due to the very low number of obtained sequences.

Sample	Number of valid bacterial sequences	Sample	Number of valid fungal sequences
Lime-1a-SP	896	Oak-1-SP*	96
Walnut-1c-SP	1110	Walnut-1a-SP*	319
Lime-1b-SP	3083	Cedar-1a-AU*	363
Walnut-3b-AU	4351	Walnut-4-AU	524
Walnut-4-AU	6242	Lime-1a-SP	715
Cedar-1b-AU	6528	Walnut-1-AU	1189
Walnut-2-AU	6615	Plane_tree-2-AU	1424
Magnolia-1-AU	6822	Magnolia-1a-SP	2001
Lime-1-AU	6907	Oak-1-AU	2118
Walnut-1-AU	7320	Magnolia-1b-SP	3354
Walnut-3-AU	7457	Walnut-1b-SP	3736
Oak-1-AU	8548	Lime-1b-SP	4226
Magnolia-1a-SP	9337	Lime-1-AU	6121
Walnut-1b-SP	9975	Plane_tree-1-AU	10090
Magnolia-1b-SP	10351	Cedar-1b-AU	14406
Plane_tree-2-AU	11027	Walnut-1c-SP	16891
Plane_tree-1-AU	11667	Magnolia-1-AU	22066
Cedar-1a-AU	18137	Walnut-3b-AU	26300
Oak-1-SP	52556	Walnut-3-AU	101218
Walnut-1a-SP	132723	Walnut-2-AU	185031

Supplementary Table 6. Relative abundance (%) of bacterial populations in tree cavity organic soil (TCOS) possessing selected hydrocarbon-degrading genes (AlkB, Cyp153: alkane hydroxylases; AlmA: flavin binding monooxygenases; LadA: long chain alkane hydroxylases; TmoE: toluene-4-monooxygenase; MAH alpha/beta: monoaromatic dioxygenases; TomA4: toluene-ortho-monooxygenase; DmpO: phenol hydroxylase; Naph: sum of NdoB and non-NdoB-type naphthalene dioxygenases; AhyA: putative alkane C2 methylene hydroxylase; CmdA: ethylbenzene dehydrogenase). See Khot et al., 2022 for details on methodology.

	**Aerobiosis**										**Anaerobiosis**	
	**Alkanes**				**Mono-aromatic**					**Poly-aromatic**	**Alkanes**	**Mono-aromatic**
**Sample**	AlkB	Cyp153	AlmA group III	LadA alpha	TmoE	MAH alpha	MAH beta	TomA4	DmpO	Naph	AhyA	CmdA
Cedar-1a-AU	10.6	4.0	1.3	15.7	0.4	1.3	0.8	0.4	1.2	11.7	0.0	0.0
Cedar-1b-AU	12.4	3.5	0.8	13.4	1.7	0.8	1.3	0.4	0.6	8.1	0.2	0.2
Magnolia-1-AU	2.0	0.9	0.1	1.9	0.1	0.1	0.1	0.2	0.0	0.6	0.7	0.6
Walnut-1-AU	7.3	1.7	1.6	16.2	0.4	1.3	1.0	0.4	1.1	12.4	0.0	0.0
Walnut-2-AU	7.7	3.1	0.0	6.9	0.5	1.1	0.6	0.6	0.1	7.2	0.0	0.0
Walnut-3-AU	9.0	3.8	0.0	4.8	0.4	0.4	1.1	0.2	0.0	4.9	2.0	2.0
Walnut-3b-AU	9.3	3.1	0.3	6.9	0.4	0.4	1.3	0.1	0.2	5.0	1.2	1.1
Walnut-4-AU	9.2	3.5	0.2	6.4	0.2	0.5	1.4	0.2	0.1	5.1	1.3	1.2
Plane_tree-1-AU	3.9	1.7	0.1	5.4	0.0	0.2	0.3	0.1	0.0	2.7	2.1	1.9

Supplementary Material

Plane_tree-2-AU	1.4	0.6	0.1	4.7	0.0	0.2	0.2	0.1	0.1	1.7	0.4	0.3
Oak-1-AU	4.6	1.2	0.0	9.0	0.1	0.6	0.7	0.0	0.1	2.5	0.4	0.4
Lime-1-AU	7.3	2.9	1.3	8.3	0.3	1.1	1.4	0.3	0.1	5.6	0.4	0.3
Magnolia-1a-SP	10.4	3.3	0.0	9.5	0.9	0.7	1.7	1.2	0.4	7.9	0.5	0.4
Magnolia-1b-SP	8.0	3.1	0.0	5.9	0.3	0.3	1.3	0.1	0.1	4.4	0.3	0.3
Walnut-1a-SP	12.1	4.9	0.1	8.1	0.3	0.3	1.0	0.2	0.2	6.3	0.5	0.4
Walnut-1b-SP	4.1	6.6	0.2	14.3	0.1	0.1	0.2	0.2	0.8	8.0	0.0	0.0
Walnut-1c-SP	5.9	1.9	0.0	8.8	0.4	0.2	0.8	1.7	0.5	6.9	0.8	0.8
Oak-1-SP	5.0	2.4	0.0	3.9	0.1	0.2	0.2	0.2	0.0	3.2	0.0	0.0
Lime-1a-SP	8.0	2.2	0.0	6.1	0.5	0.4	1.8	0.7	0.2	4.9	0.0	0.0
Lime-1b-SP	7.3	3.2	0.0	4.6	0.3	0.2	1.1	0.4	0.1	4.0	0.8	0.8
